# Single-Molecule Traps in Covalent Organic Frameworks for Selective Capture of C_2_H_2_ from C_2_H_4_-Rich Gas Mixtures

**DOI:** 10.34133/research.0458

**Published:** 2024-08-26

**Authors:** Yilun Zhou, Yinghui Xie, Xiaolu Liu, Mengjie Hao, Zhongshan Chen, Hui Yang, Geoffrey I. N. Waterhouse, Shengqian Ma, Xiangke Wang

**Affiliations:** ^1^ College of Environmental Science and Engineering, North China Electric Power University, Beijing 102206, P.R. China.; ^2^ MacDiarmid Institute for Advanced Materials and Nanotechnology, School of Chemical Sciences, The University of Auckland, Auckland 1142, New Zealand.; ^3^ Department of Chemistry, University of North Texas, Denton, TX 76201, USA.

## Abstract

Removing trace amounts of acetylene (C_2_H_2_) from ethylene (C_2_H_4_)-rich gas mixtures is vital for the supply of high-purity C_2_H_4_ to the chemical industry and plastics sector. However, selective removal of C_2_H_2_ is challenging due to the similar physical and chemical properties of C_2_H_2_ and C_2_H_4_. Here, we report a “single-molecule trap” strategy that utilizes electrostatic interactions between the one-dimensional (1D) channel of a covalent organic framework (denoted as COF-1) and C_2_H_2_ molecules to massively enhance the adsorption selectivity toward C_2_H_2_ over C_2_H_4_. C_2_H_2_ molecules are immobilized via interactions with the O atom of C=O groups, the N atom of C≡N groups, and the H atom of phenyl groups in 1D channels of COF-1. Due to its exceptionally high affinity for C_2_H_2_, COF-1 delivered a remarkable C_2_H_2_ uptake of 7.97 cm^3^/g at 298 K and 0.01 bar, surpassing all reported COFs and many other state-of-the-art adsorbents under similar conditions. Further, COF-1 demonstrated outstanding performance for the separation of C_2_H_2_ and C_2_H_4_ in breakthrough experiments under dynamic conditions. COF-1 adsorbed C_2_H_2_ at a capacity of 0.17 cm^3^/g at 2,000 s/g when exposed to 0.5 ml/min C_2_H_4_-rich gas mixture (99% C_2_H_4_) at 298 K, directly producing high-purity C_2_H_4_ gas at a rate of 3.95 cm^3^/g. Computational simulations showed that the strong affinity between C_2_H_2_ and the single-molecule traps of COF-1 were responsible for the excellent separation performance. COF-1 is also robust, providing a promising new strategy for the efficient removal of trace amounts of C_2_H_2_ in practical C_2_H_4_ purification.

## Introduction

Ethylene (C_2_H_4_) is the most important product in the petrochemical industry, finding widespread use in the manufacture of organic chemicals and polymers [1,2]. However, industrial C_2_H_4_ production processes, such as alkane dehydrogenation and naphtha cracking, typically create trace amounts of acetylene (C_2_H_2_) as a by-product [1]. Removal of C_2_H_2_ from C_2_H_4_ feedstocks is of enormous economic and practical importance, since trace C_2_H_2_ can adversely impact polymerizations and synthetic applications that require high-purity C_2_H_4_ [3,4]. Cryogenic distillation (also known as low-temperature rectification) is a widely used commercial separation process involving liquefying gas mixtures at very low temperatures (183 to 258 K with 7 to 28 bar) and then selectively distilling a specific gas component at its boiling point [5]. This method is frequently applied to separate C_2_H_2_ and C_2_H_4_, but is expensive and energy intensive. To avoid such high-energy consumption and operating costs, physical adsorption methods using porous solid adsorbent have been widely explored in recent years. Porous materials such as zeolites [6,7], carbons [8,9], and MXenes [10,11] have all been studied as adsorbents to remove C_2_H_2_ from C_2_H_4_-rich gas mixtures, but their separation processes are generally unsatisfactory due to poor selectivity and a low adsorption capacity. Metal-organic frameworks (MOFs) demonstrate promise in C_2_H_2_/C_2_H_4_ separations, but suffer stability issues [12–19].

Recently, covalent organic frameworks (COFs) have garnered a great deal of attention for hydrocarbon separations, such as CO_2_/CH_4_ [20–26], C_2_H_4_/C_2_H_6_ [27–32], and C_2_H_4_/C_3_H_6_ [33], due to their diverse structures, high specific surface areas, programmable pore characteristics, high stability, and other features [34]. Taking advantages of the COF framework’s differing affinity for C_2_H_2_ and C_2_H_4_, COFs have been designed to separate C_2_H_2_ from C_2_H_4_ [35–41]. However, few studies relating to the dynamic removal of trace amounts of C_2_H_2_ from C_2_H_4_-rich gas mixtures have been reported [2]. Jiang and co-workers [41] described a robust porous aromatic framework (PAF-110), which exhibited a moderate C_2_H_2_ selectivity of 3.9 at 298 K and 1 bar. Next, Wang and co-workers [29] utilized a modulator-assisted strategy to control the interlayer stacking in an imide-linked 2D COF. As-synthesized ABC stacking COF led to a 60% increase in volumetric C_2_H_2_ uptake compared with the AA stacking. Subsequently, an olefin-linked COF was synthesized, with a pore size and chemical environment suitable for trapping C_2_H_2_ preferentially over C_2_H_4_ [42]. These pioneering works take advantage of the pore size, pore dimensions, and location of electron-donating atoms (such as N and O) to selectively coordinate C_2_H_2_, thus achieving good separation of C_2_H_2_ and C_2_H_4_. However, many other factors affect the separation efficiency of C_2_H_2_ and C_2_H_4_, with the specific nature of the interactions between the guest gas molecules and host adsorption sites in COFs being poorly understood at a molecular level, warranting further exploration. Furthermore, achieving a high separation efficiency at low pressures is typically challenging for COFs. Therefore, it is of great importance to discover COF-based adsorbents capable of separating C_2_H_2_ and C_2_H_4_ under practical conditions, especially at low C_2_H_2_ pressures.

**Fig. 1.**
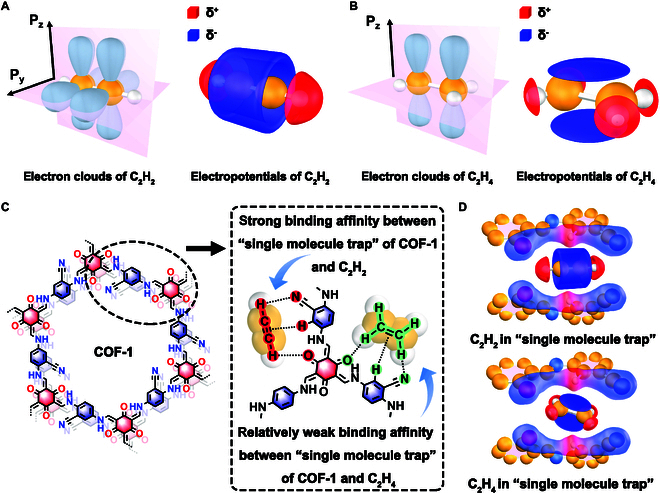
(A) Electron clouds and electron potentials of C_2_H_2_. (B) Electron clouds and electron potentials of C_2_H_4_. (C and D) Structure of COF-1 and a schematic showing the interaction between single-molecule traps in COF-1 with C_2_H_2_ and C_2_H_4_. Clouds in red represent relative positive potential, and clouds in blue represent relative negative potential in (A), (B), and (D).

The distribution of electron clouds in C_2_H_2_ gives the molecule positive–negative–positive electronegativity and high rotational symmetry along its molecular axis (Fig. 1A and Table S1). The same rotational symmetry does not exist in the C_2_H_4_ molecule. As such, the charge distribution along the C–H bonds in C_2_H_4_ is not as extreme as in C_2_H_2_ (Fig. 1B and Table S1). We believe that suitable adsorption sites can strengthen the separate effect [43]. Based on the differences in electropositivity at the hydrogen atoms in each molecule, we designed and synthesized a robust COF (denoted as COF-1) for the selective separation of C_2_H_2_ and C_2_H_4_. Owing to the action of C=O, C≡N, and C–H (from the phenyl ring) in the framework, which acted synergistically to form single-molecule traps for C_2_H_2_, COF-1 exhibited a record-high static C_2_H_2_ adsorption capacity (7.97 cm^3^/g) at 298 K at ultralow pressures (0.01 bar), outperforming all previously reported COF adsorbents. Dynamic breakthrough experiments showed that COF-1 takes up 0.17 cm^3^/g of C_2_H_2_ at 2,000 s/g in a C_2_H_2_/C_2_H_4_ = 1:99 (v/v) gas mixture at 298 K. This enables the direct production of 3.95 cm^3^/g (0.27 ml/min/g) high-purity C_2_H_4_ gas under 0.5 ml/min inlet C_2_H_4_-rich gas mixture (99% C_2_H_4_) at 298 K and 1 bar, comparable to other state-of-the-art adsorbents. Molecular simulations based on the grand canonical Monte Carlo (GCMC), together with first-principles density functional theory (DFT) calculations, revealed that lone pairs of sp hybridized nitrogen atoms in C≡N groups and lone pairs of sp^2^ hybridized oxygen atoms in C=O groups in the COF-1 framework exhibited negative potentials, while the H atoms in phenyl ring in the framework exhibited positive potentials, with the resulting negative(δ^−^)–positive(δ^+^)–negative(δ^−^) charge distribution being perfect for trapping positive(δ^+^)–negative(δ^−^)–positive(δ^+^) charged C_2_H_2_ (Fig. 1C and D). In contrast, C_2_H_4_ exhibited a relatively low affinity for the single-molecule traps in COF-1, as expected owing to the differing structure, size, and charge arrangement in C_2_H_4_ (Fig. 1C and D). The structure–property relationships developed in this work allowed us to pinpoint the factors influencing binding affinity of COF-1 toward each gas molecule and ultimately explain the C_2_H_2_/C_2_H_4_ separation performance, guiding the future design of COF-based adsorbents with multiple cooperative functionalities for selective gas separations.

## Results and Discussion

### COF synthesis and characterization

**Fig. 2.**
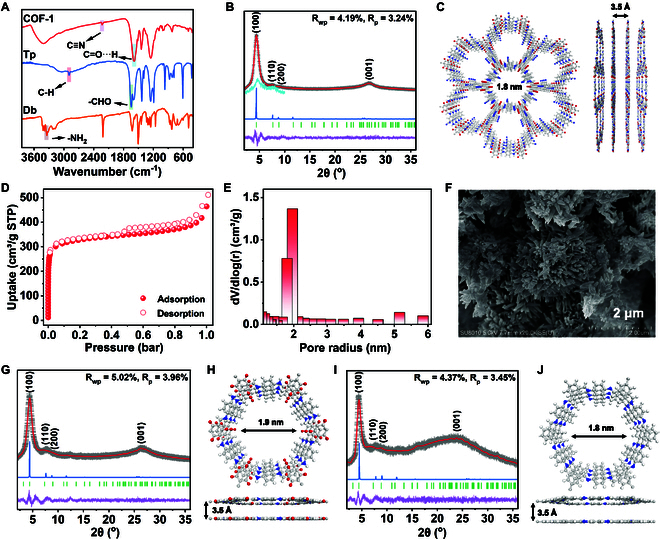
(A) FT-IR spectra of COF-1 and related linkers. (B) PXRD patterns of COF-1 with refinement. Pawley refinement, simulated results, and Bragg positions are in red, blue, and green, respectively. Experimental data (gray cross) display little differences (purple) with simulation. SAXS data were used for zero-shift correction in (B). (C) Top and side views of the corresponding refined structure of COF-1. (D and E) N_2_ adsorption/desorption isotherms of COF-1 and corresponding pore size distribution measured at 77 K. (F) SEM image of COF-1. (G) PXRD patterns of COF-2 with refinement. Pawley refinement, simulated results, and Bragg positions are in red, blue, and green, respectively. Experimental data (gray cross) display little differences (purple) with simulation. (H) Top and side views of the corresponding refined structure of COF-2. (I) PXRD patterns of COF-3 with refinement. Pawley refinement, simulated results, and Bragg positions are in red, blue, and green, respectively. Experimental data (gray cross) display little differences (purple) with simulation. (J) Top and side views of the corresponding refined structure of COF-3.

We proposed that “single-molecule traps” could be used to remove trace amounts of C_2_H_2_ in C_2_H_2_/C_2_H_4_ gas mixtures. For this purpose, we first synthesized COF-1 by reacting 1,3,5-triformylphloroglucinol (Tp) and 2,5-diaminobenzonitrile (Db) in a mixture of mesitylene/1,4-dioxane/acetic acid at 120 °C for 72 h. The Fourier transform infrared (FT-IR) spectrum of COF-1 showed the disappearance of the -CHO groups of Tp at 1,644 cm^−1^ and -NH_2_ groups of Db signal at 3,348 cm^−1^, indicating the successful Schiff-base condensation between Tp and Db (Fig. 2A) [44,45]. No C=N signals were observed, but an intense C=O···H stretch around 1,587 cm^−1^ appeared due to imine groups transformed to β-ketamine moieties in COF-1. The COF-1 product also showed an absorption peak at 2,225 cm^−1^ due to C≡N stretching of cyano groups on the aromatic rings of the Db linker (Fig. 2A) [46]. The cyano groups were thus retained with the formation of COF-1. The thermogravimetric analysis (TGA) demonstrated that COF-1 displayed ~10% weight loss up to approximately 400 °C under a N_2_ atmosphere, suggesting good thermostability (Fig. S1). The crystalline structure of COF-1 was further experimentally and theoretically determined by powder x-ray diffraction (PXRD), small-angle X-ray scattering (SAXS), and theoretical simulations. The experimental PXRD pattern (SAXS used for zero-shift correction) showed diffraction peaks at 2θ angle around 4.4°, 7.9°, 8.9°, and 26.8°, which could be assigned to the (100), (110), (200), and (001) planes, respectively (Fig. 2B). Pawley refinements were performed on the experimental PXRD data to yield unit cell parameters of *a* = *b* = 23.29 Å, *c* = 3.49 Å, *α* = *β* = 90°, *γ* = 120° (residuals *R*_p_ = 3.24% and *R*_wp_ = 4.19%) for COF-1 (Table S2). The eclipsed stacking (AA) mode was constructed with the unit cell parameter described above to generate a simulated diffraction pattern for comparison with the experimental data. The experimental and simulated data matched well, showing a 3.5-Å layer spacing (Fig. 2C). The hexagonal pore diameter is around ~1.8 nm (Fig. 2C).

N_2_ sorption isotherms were measured at 77 K for COF-1 to investigate its porosity (Fig. 2D and Fig. S2). The adsorption–desorption isotherms showed type I/IV curves, which suggested microporous structures. The Brunauer–Emmett–Teller (BET) surface area and total pore volume of COF-1 were determined to be 997 m^2^/g and 0.71 cm^3^/g, respectively. The pore size analysis revealed that the average pore diameter is ~1.89 nm, well in line with the predicted pore diameter for AA stacking geometries of the framework (Fig. 2E). The scanning electron microscopy (SEM) image of COF-1 showed a flower-like morphology composed of nanorods (Fig. 2F). The transmission electron microscopy (TEM) and high-resolution TEM (HRTEM) images confirmed the flower-like morphology, with lattice fringe spacings of 1.96 nm (corresponding to 100 lattice plane) consistent with the porous structures measured by PXRD analysis (Fig. S3).

To validate the utility of using the O atom of C=O, N atom of C≡N, and H atom of phenyl rings to create “single-molecule traps” for separation of C_2_H_2_/C_2_H_4_, we also synthesized structural analogs (COF-2 and COF-3) for comparison. The synthesis and characterization data for COF-2 and COF-3 are provided in Fig. 2G to J and the Supplementary Materials (Tables S3 and S4 and Figs. S4 to S6). Briefly, COF-2 was synthesized using the same general protocol as COF-1 by condensation of Tp with p-phenylenediamine (Pa). COF-3 was synthesized by condensation of Pa and 1,3,5-benzenetricarboxaldehyde (TFB) via similar synthetic routes. Compared to COF-1, COF-2 lacks the C≡N groups in the one-dimensional (1D) channels of the framework, while COF-3 was deficient in both C=O and C≡N groups in the 1D channels (Fig. 2H and J).

### C_2_H_2_ and C_2_H_4_ sorption performance

**Fig. 3.**
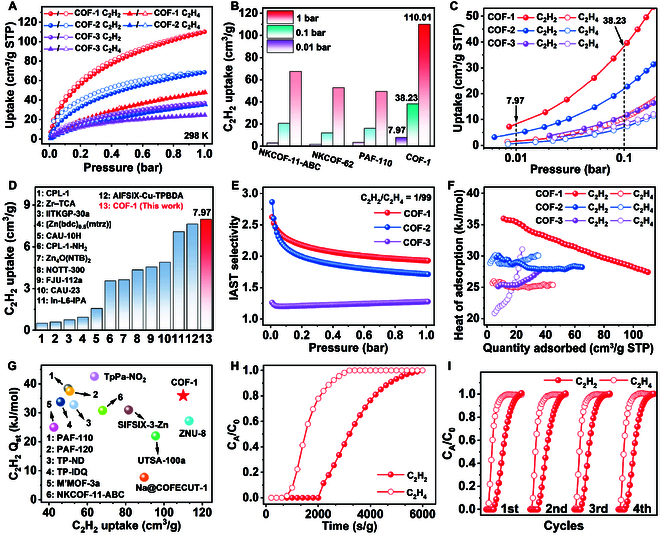
(A) Single-component C_2_H_2_ and C_2_H_4_ adsorption (filled symbols) and desorption (open symbols) isotherms of COF-1 (red), COF-2 (blue), and COF-3 (purple) at 298 K. (B) Comparison of C_2_H_2_ adsorption capacity at 298 K (0.01, 0.1, and 1 bar) of COF-1 with reported top-performing COFs. (C) Expanded view of the low-pressure single-component C_2_H_2_ and C_2_H_4_ adsorption isotherms of COF-1, COF-2, and COF-3 at 298 K. (D) Comparison of C_2_H_2_ adsorption capacity of COF-1 with reported top-performing MOFs at 298 K and 0.01 bar. (E) IAST selectivity of C_2_H_2_/C_2_H_4_ (1:99, v/v) mixture for COF-1, COF-2, and COF-3 at 298 K. (F) *Q*_st_ for C_2_H_2_ and C_2_H_4_ adsorption in COF-1, COF-2, and COF-3. (G) Comparison of *Q*_st_ values and uptakes of C_2_H_2_ at 298 K and 1 bar among top-performing COFs and MOFs. (H) Experimental breakthrough curves of C_2_H_2_/C_2_H_4_ (1:99, v/v) mixtures for COF-1 at a flow rate of 0.5 ml/min at 298 K (1 bar). (I) Cycle breakthrough curves of C_2_H_2_/C_2_H_4_ (1:99, v/v) mixtures for COF-1 at a flow rate of 0.5 ml/min.

We first performed the single-component C_2_H_2_ and C_2_H_4_ sorption measurements on COF-1, COF-2, and COF-3 at 298 and 273 K to evaluate their adsorption performance (Figs. 7 to S10). The C_2_H_2_ adsorption capacities of COF-1, COF-2, and COF-3 were 110.01, 68.49, and 36.89 cm^3^/g at 298 K and 1 bar, respectively (Fig. 3A). The uptake capacity of COF-1 at 298 K and 1 bar is notably higher than that of other benchmark adsorbents such as NKCOF-11-ABC (68 cm^3^/g) [29], PAF-110 (49.95 cm^3^/g) [41], PAF-120 (50.85 cm^3^/g) [39], Na@COF-ECUT-1 (89.7 cm^3^/g) [38], TpPa-NO_2_ (63.73 cm^3^/g) [35], TP-ND (53 cm^3^/g) [36], and USTB-25-3D (57.7 cm^3^/g) [47]. Next, the adsorption performance of the COFs at ultralow pressures of C_2_H_2_ was investigated. The C_2_H_2_ uptake capacities (298 K) of COF-1 at ultralow pressure (0.01 bar) and low pressure (0.1 bar) were 7.97 and 38.23 cm^3^/g, respectively, implying a strong affinity between single-molecule traps and C_2_H_2_ (Fig. 3B and C and Table S5) [29,41,42]. To the best of our knowledge, such high uptakes at such ultralow pressure have not been reported in other COF adsorbents. Moreover, these numbers even exceed some reported state-of-the-art MOFs under similar conditions (Fig. 3D and Table S6) [15,48–56]. COF-2 and COF-3 showed much lower C_2_H_2_ adsorption capacities under similar pressures. Moreover, the uptake of C_2_H_4_ at 298 K and 1 bar for COF-1, COF-2, and COF-3 were 42.7, 35.4, and 24.5 cm^3^/g (Fig. 3A). The adsorption capacities of COF-1 for C_2_H_4_ at 298 K at 0.01 and 0.1 bar were 1.56 and 10.71 cm^3^/g, respectively, which were all much lower than the adsorption capacities for C_2_H_2_ under the same temperature and pressure conditions (Fig. 3C). Taken together, the performance of COF-1 and COF-2 shows that the introduction of C≡N groups in the 1D channels significantly improved the C_2_H_2_ adsorption capacity, while the C_2_H_4_ uptake barely changed. When both C=O and C≡N are absent (i.e., COF-3), C_2_H_2_ and C_2_H_4_ were adsorbed in almost the same amount. Clearly, the synergistic action of C=O and C≡N groups in the 1D channels of COF-1 created “single-molecule traps” with fast adsorption and high selectivity for C_2_H_2_.

Motivated by the high uptake and preferential binding of C_2_H_2_ by COF-1, the adsorption selectivity was estimated on the basis of ideal adsorbed solution theory (IAST) using a C_2_H_2_/C_2_H_4_ (1:99, v/v) gas mixture at 298 K. The predicted C_2_H_2_/C_2_H_4_ (1:99, v/v) selectivity of COF-1 was 2.6 at 298 K (Fig. 3E). COF-2 and COF-3 not possessing single-molecule traps had lower predicted IAST selectivities, particularly COF-3, which was lacking in suitable functional groups for binding C_2_H_2_. This explains why COFs devoid of suitable functional modifications have difficulty separating C_2_H_2_ and C_2_H_4_ (i.e., molecules with similar physical and chemical structures). The coverage-dependent isosteric heat of adsorption (*Q*_st_) calculated using the Clausius–Clapeyron equation was used to evaluate the affinities of each COF toward C_2_H_2_ and C_2_H_4_ (Fig. 3F and Figs. S11 to S16) [57,58]. COF-1 exhibited a *Q*_st_ of 35.95 kJ/mol at near-zero loading for C_2_H_2_, while the *Q*_st_ value was 25.68 kJ/mol for C_2_H_4_. The data indicate a preferred affinity toward C_2_H_2_ over C_2_H_4_ (Fig. 3F). Moreover, the *Q*_st_ curve of C_2_H_2_ showed a decrease with increasing C_2_H_2_ uptake, suggesting that adsorption sites with higher interaction energy were first occupied before sites with lower energy. COF-2 showed similar *Q*_st_ values for both gases and flat curves, suggesting poor adsorption selectivity. The gradually increasing *Q*_st_ curves for C_2_H_2_ adsorption by COF-3 were attributed to attractive interactions between the adsorbed molecules, indicating that the molecule–framework interactions were weaker than the molecule–molecule interactions. The steeper rising curve for C_2_H_4_ showed that almost all adsorption behavior in COF-3 came from interactions between C_2_H_4_ molecules instead of the C_2_H_4_–COF interactions that we expected. The respective *Q*_st_ values for the different COFs were obviously related to the functional groups in the 1D channels, with C≡N and C=O groups in COF-1 greatly enhancing the C_2_H_2_ adsorption affinity, which was also reflected in single-component adsorption isotherms and IAST results. On the basis of these results, the “single-molecule traps” in COF-1 offered well-fitting adsorption sites for C_2_H_2_, which acted to enhance the host–guest interactions between the framework and C_2_H_2_ molecules. COF-1 displayed a high C_2_H_2_ uptake and a moderate C_2_H_2_ *Q*_st_ value, the latter indicating that a low energy input would be required to recover the adsorbed C_2_H_2_ (Fig. 3G and Table S8) [15,17,18,29,35,36,38,39,41,59]. These results identified COF-1 as a very promising candidate for C_2_H_2_/C_2_H_4_ separations, including the capture of trace amounts of C_2_H_2_.

### C_2_H_2_/C_2_H_4_ breakthrough tests

We next carried out breakthrough experiments under dynamic conditions to examine the practical C_2_H_2_/C_2_H_4_ separation performance of COF-1. Initially, 0.45 g of COF-1 was packed into a fixed adsorbent bed, and breakthrough experiments were conducted at room temperature (298 K) using a C_2_H_2_/C_2_H_4_ (1:99, v/v) gas mixture with a flow rate of 0.5 ml/min. As shown in Fig. 3H, C_2_H_4_ first eluted through the column at 800 s/g, while C_2_H_2_ did not reach saturation until 2,000 s/g. The dynamic adsorption capacity values of COF-1 for C_2_H_2_ were calculated to be 0.26 cm^3^/g, allowing 3.95 cm^3^/g (average delivery of 0.27 ml/min/g) of high-purity C_2_H_4_ gas under 0.5 ml/min inlet C_2_H_4_-rich gas mixture (99% C_2_H_4_) at 298 K and 1 bar. These results revealed that COF-1 could efficiently bind C_2_H_2_ molecules to yield pure C_2_H_4_ under dynamic conditions. Next, further breakthrough experiments were performed using 1:1 (v/v, 1.0 ml/min) C_2_H_2_/C_2_H_4_ mixtures (Fig. S17). Under these conditions, C_2_H_4_ breakthrough occurred first at 250 s/g, followed by C_2_H_2_ at 2,430 s/g. The calculated selectivity is ~3, thus maintaining a preferential adsorption of C_2_H_2_ over C_2_H_4_. Additionally, cycling dynamic breakthrough experiments using C_2_H_2_/C_2_H_4_ (1:99, v/v) and a flow rate of 0.5 ml/min were conducted to assess the recyclability of COF-1. The breakthrough time was almost unchanged after four continuous cycles, indicating that COF-1 can be easily regenerated and recycled without any significant loss in separation performance (Fig. 3I). Simultaneously, the crystallinity of COF-1 was retained under various treatments for 24 h (Fig. S18). PXRD results further revealed that the crystallinity of COF-1 was retained after the breakthrough experiments, suggesting good stability and long-term durability (Fig. S19). The data verified the robustness of COF-1 as a selective adsorbent for C_2_H_2_.

### Mechanism study

**Fig. 4.**
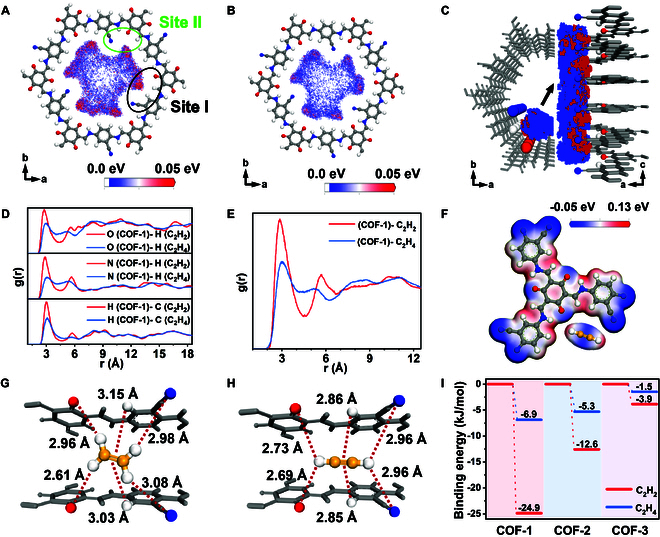
Computational simulations showing the density distribution of (A) C_2_H_2_ and (B) C_2_H_4_ on COF-1 at 298 K and 1 bar. (C) Computational simulations density distribution for the dynamic separation of C_2_H_2_/C_2_H_4_ (1:99, v/v) at 298 K and 1 bar. Red points represent C_2_H_2_, and blue points represent C_2_H_4_. (D) RDF plots showing the contributions of various atoms to the adsorption of C_2_H_2_ and C_2_H_4_ in COF-1 at 298 K and 1 bar. (E) Summarized RDF plots for C_2_H_2_ and C_2_H_4_ adsorption in COF-1 at 298 K and 1 bar. (F) Electrostatic potentials of site I in COF-1 and C_2_H_2_. The DFT optimized gas adsorption configuration and interaction distances of (G) C_2_H_2_ and (H) C_2_H_4_ in COF-1. (I) Binding energies of COF-1, COF-2, and COF-3 with C_2_H_2_ and C_2_H_4_.

GCMC simulations, molecular dynamics (MD) simulations, and DFT calculations were performed to gain deeper insights into the mechanism of selective adsorption of C_2_H_2_ over C_2_H_4_ in COF-1. The single-component adsorption simulation by COF-1 at 298 K and 1 bar showed that C_2_H_2_ molecules were mainly adsorbed at site I (black ellipse in Fig. 4A) in the 1D channels (between the N atom of C≡N and O atom of C=O), which represented the best-fitting configuration for the single-molecule traps (Fig. 4A). Adsorption site II (green ellipse in Fig. 4A) in COF-1 contained N, O, and H atoms but showed a weaker binding affinity toward C_2_H_2_ (Fig. 4A). In contrast, the weaker density distribution for C_2_H_4_ in both site I and site II further demonstrated that COF-1 displayed a stronger adsorption affinity toward C_2_H_2_ than C_2_H_4_ when compared at the same density distribution scale (Fig. 4B). COF-2 and COF-3 showed negligible difference in C_2_H_2_ and C_2_H_4_ adsorption at the same scale (Figs. S20 and S21). It can therefore be concluded that the presence of both C≡N or C=O groups in the 1D channels of COF-1 acts synergistically to selectively capture C_2_H_2_.

Subsequently, GCMC calculations were used to predict the molecule distribution during the dynamic separation of C_2_H_2_/C_2_H_4_ (1:99, v/v) at 298 K and 1 bar (Fig. 4C). C_2_H_2_ molecules occupied the main adsorption sites in COF-1 even when the C_2_H_2_ content was only 1 vol.% (red color parts), suggesting a strong binding affinity toward C_2_H_2_. Meanwhile, the C_2_H_4_ molecules were distributed elsewhere (blue color parts). These binary gas adsorption simulations supported the idea that COF-1 showed an extraordinary C_2_H_2_ affinity compared to C_2_H_4_ at ultralow pressures, in perfect accord with the experimental results. The proper layer distance in COF-1 together with C=O, C≡N, and C–H (phenyl) groups in the 1D channels afforded effective single-molecule traps for C_2_H_2_. Radial distribution function (RDF) analysis verified a strong affinity between the target atoms in COF-1 for C_2_H_2_ molecules, evidenced by a number of *g*(*r*) peaks. As shown in Fig. 4D and E, the *g*(*r*) peak intensity of C_2_H_2_ was obviously higher than that of C_2_H_4_, further supporting the strong affinity between the single-molecule traps in COF-1 and C_2_H_2_.

DFT calculations were next conducted on different interaction models. The layer distance of COF-1 is ~3.5 Å, which was slightly larger than the molecule size of C_2_H_2_ (5.5 Å × 3.3 Å × 3.3 Å). The model shows that C_2_H_2_ was preferentially located in the single-molecule trap (site I) with a microenvironment of negative(δ^−^)–positive(δ^+^)–negative(δ^−^) charge distribution formed by the O atom of C=O, N atom of C≡N, and H atom of a phenyl ring. C_2_H_2_ molecules displayed positive(δ^+^)–negative(δ^−^)–positive(δ^+^) electronegativity and highly rotational symmetry. Specifically, O atoms and N atoms possessing a negative charge distribution (δ^−^) interacted with the H atom of C_2_H_2_ (δ^+^) from two directions, with an H atom of the phenyl ring possessing positive charge distribution (δ^+^) interacting with the π electrons (δ^−^) of C_2_H_2_ (Fig. 4F). Each C_2_H_2_ molecule is bonded by two nitrogen atoms from C≡N···H (2.96 Å), two O atoms from C=O···H (2.69 Å to 2.73 Å), and H atoms through C–H···π (2.85 Å to 2.86 Å) in site I (Fig. 4G). The molecule size of C_2_H_4_ (4.8 Å × 4.1 Å × 3.2 Å) is slightly larger than that of C_2_H_2_, which impedes adsorption in the 1D channels due to the steric effects (Fig. 4H). Moreover, the charge distribution in C_2_H_4_ is not well matched to COF-1 like it was for C_2_H_2_, leading to specific directional interactions that impede adsorption (Fig. S22). Site II in COF-1, having two N atoms and two O atoms on the same side of the adsorbing gas molecules, displayed weaker binding affinity toward both C_2_H_2_ and C_2_H_4_ (Figs. S23 and 24). Taken together, the strong host–guest interactions of COF-1 with C_2_H_2_ are in accord with experimental observations. Although site II of COF-1 was not ideal for capture of C_2_H_2_, it still favored C_2_H_2_ over C_2_H_4_ and thus helped to remove more C_2_H_2_. COF-2 and COF-3 showed little difference in their binding affinities for C_2_H_2_ and C_2_H_4_ due to the deficiency of C_2_H_2_-specific adsorption sites (Figs. S25 to 28). The calculated static binding energy of site I in COF-1 for C_2_H_2_ is −24.9 kJ/mol, compared with −6.9 kJ/mol for C_2_H_4_ at the same site (Fig. 4I). The calculated binding energies for C_2_H_2_/COF-2, C_2_H_4_/COF-2, C_2_H_2_/COF-3, and C_2_H_4_/COF-3 are −12.6, −5.3, −3.9, and −1.5 kJ/mol, respectively. These results further explain why COF-2 and COF-3 delivered inferior C_2_H_2_ adsorption capacities and poor selectivity for C_2_H_2_/C_2_H_4_ separations compared to COF-1.

## Conclusion

In summary, COF-1 is one of the best adsorbents developed to date for C_2_H_4_, capable of selectively adsorbing trace amounts of C_2_H_2_ under ambient conditions. The single-molecule traps in COF-1 are complementary to C_2_H_2_ in size and electrostatic potentials, allowing noncovalent interactions to trap guest C_2_H_2_ molecules. C_2_H_4_ molecules do not bind efficiently to the single-molecule traps in COF-1. Therefore, COF-1 offers a very high affinity for C_2_H_2_ at ultralow pressures, favoring the capture of C_2_H_2_ in binary C_2_H_2_/C_2_H_4_ gas mixtures. Our findings are relevant to the practical challenges of purifying C_2_H_4_ and industrial feedstocks, revealing an obvious structure–separation performance relationship along with mechanistic understanding at the molecular level. This work shows that the synergistic action of specific functional groups in the 1D channels of COFs can be harnessed to remove trace amounts of C_2_H_2_ from industrial C_2_H_4_-rich gas mixtures, thus delivering high-purity industrial C_2_H_4_ for various applications.

## Materials and Methods

### Materials and measurements

The reagents and solvents used in this study were sourced from commercial suppliers and used without further purification. Tp, TFB, and Pa were purchased from Jilin Chinese Academy of Sciences–Yanshen Technology Co. Ltd. Db was purchased from Bide Pharmatech Co. Ltd. Mesitylene, 1,4-dioxane, and acetic acid were purchased from Shanghai Macklin Biochemical Technology Co. Ltd. Ultrapure water was obtained from a Millipore system (18.25 MΩ·cm). FT-IR spectra were collected on a SHIMADZU IRTracer-100. TGA analyses were carried out on a NETZSCH STA 2500 instrument and Rigaku TG/DTA 8122. PXRD patterns were recorded on a Cu Kα source Rigaku SmartLab SE X-ray diffractometer. SAXS data were collected on a Rigaku SmartLab SE for a zero-shift correction. SEM images were recorded on a TESCAN MIRA4 SEM. TEM and HRTEM images were recorded on a FEI Talos F200x TEM. BET surface areas were determined from N_2_ adsorption/desorption isotherms collected at 77 K on a Micromeritics ASAP 2020 plus. Pore size distributions were obtained from the adsorption isotherms using a Barrett–Joyner–Halenda (BJH) method. Adsorption isotherms for C_2_H_2_ and C_2_H_4_ were collected at 273 and 298 K on a Micromeritics ASAP 2020 plus. A temperature-programmed water bath was used to maintain temperatures of 273 and 298 K during the adsorption experiments. Breakthrough experiments utilized a C_2_H_2_/C_2_H_4_ (1:99, v/v) gas mixture, with gases being monitored by gas chromatography (SHIMADZU Nexis GC-2030 equipped with a thermal conductivity detector). For breakthrough experiments using a C_2_H_2_/C_2_H_4_ (50:50, v/v) gas mixture, gases were monitored using a BeiShiDe Multi-component Adsorption Breakthrough Curve Analyzer (BSD-MAB).

### Syntheses

Tp (16.8 mg) and Db (16.0 mg) were dissolved in a 0.5-ml mesitylene/0.5-ml 1,4-dioxane mixed solvent solution with 0.1 ml of acetic acid (6 M) in a 5-ml glass tube. After sonication for 30 min, the mixture was frozen in a liquid nitrogen bath. Next, glass tube was flame sealed with a gas torch. Sealed tube was heated at 120 °C for 3 d, and the solid product was collected by filtration. The as-synthesized powder was washed with tetrahydrofuran and methanol. Finally, the product was dried under vacuum at 40 °C. The obtained dark red powder is denoted herein as COF-1.

Tp (16.8 mg) and Pa (13.0 mg) were dissolved in a 0.5-ml mesitylene/0.5-ml 1,4-dioxane mixed solvent solution with 0.1 ml of acetic acid (6 M) in a 5-ml glass tube. After sonication for 30 min, the mixture was frozen in a liquid nitrogen bath. Next, glass tube was flame sealed with a gas torch. Sealed tube was heated at 120 °C for 3 d, and the solid product was collected by filtration. The as-synthesized powder was washed with tetrahydrofuran and methanol. Finally, the product was dried under vacuum at 40 °C. The obtained light red powder is denoted herein as COF-2.

TFB (13.0 mg) and Pa (13.0 mg) were dissolved in a 0.67-ml mesitylene/0.33-ml 1,4-dioxane mixed solvent solution with 0.2 ml of acetic acid (6 M) in a 5-ml glass tube. After sonication for 30 min, the mixture was frozen in a liquid nitrogen bath. Next, glass tube was flame sealed with a gas torch. Sealed tube was heated at 120 °C for 3 d, and the solid product was collected by filtration. The as-synthesized powder was washed with tetrahydrofuran and methanol. Finally, the product was dried under vacuum at 40 °C. The obtained pale yellow powder is denoted herein as COF-3.

### Fitting of adsorption isotherms for pure C_2_H_2_ and C_2_H_4_

Unary adsorption isotherms for C_2_H_2_ and C_2_H_4_ on COF-1, COF-2, and COF-3 were measured at 273 and 298 K, and then fitted using a dual-site Langmuir model.

$$q=\frac{q_{\mathrm{sat},A}b_{A}P^{\frac{1}{t_{A}}}}{1+b_{A}P^{\frac{1}{t_{A}}}}+\frac{q_{\mathrm{sat},B}b_{B}P^{\frac{1}{t_{B}}}}{1+b_{B}P^{\frac{1}{t_{B}}}}$$

where *P* is the pressure (in Pa) of the bulk gas at equilibrium with the adsorbed phase, *q* is the adsorbed amount per mass of adsorbent, *q_sat, A_* and *q_sat, B_* are the saturation capacities, *b_A_* and *b_B_* are the affinity coefficients, and 1/*t_A_* and 1/*t_B_* represent the deviations from the ideal homogeneous surface. The fitting parameters were displayed in Tables S8 to S10.

$$b_{A}=b_{A_{0}}e^{\frac{E_{A}}{\mathrm{RT}}},b_{B}=b_{B_{0}}e^{\frac{E_{B}}{\mathrm{RT}}}$$

*E_A_* and *E_B_* are the energy parameters associated. *b_A_* and *b_B_* are both temperature-dependent.

### Isosteric heat of adsorption

The binding energies of C_2_H_2_ and C_2_H_4_ were estimated using the isosteric heat of adsorption. *Q*_st_ is defined as

$$Q_{\mathrm{st}}=\left(RT^{2}\right)\frac{d\text{ln}P}{\mathrm{dT}}$$

The calculations are based on the use of the Clausius–Clapeyron equation, where 𝑃 is the pressure (in Pa), *T* is the temperature (in K), and *R* is the gas constant.

### IAST calculations of adsorption selectivity

The adsorption selectivity of C_2_H_2_/C_2_H_4_ (1:99, v/v) was established using the ideal adsorption solution theory (IAST). Adsorption selectivity is defined as follows:

$$S_{\mathrm{abs}}=\frac{x_{A}/x_{B}}{y_{A}/y_{B}}$$

where *x_A_* and *x_B_* are the equilibrium adsorption capacity, and *y_A_* and *y_B_* are the molar fractions of components A and B in the gas phase.

### Breakthrough experiments

C_2_H_2_/C_2_H_4_ = 1:99, v/v:

In a typical experiment, 450 mg of adsorbent (in the column Ø 6 mm × 400 mm) was first activated at 353 K overnight under a He flow (10 ml/min). The column containing the adsorbent was then cooled to 298 K, whereupon a C_2_H_2_/C_2_H_4_ mixture (1:99, v/v) was introduced at a flow rate of 0.5 or 1.0 ml/min, with the gas flow rate controlled using a mass flow controller. The outlet gas from the column was continuously monitored using gas chromatography (Nexis GC-2030, SHIMADZU). To evaluate the reusability of the adsorbent, the adsorbent was regenerated in situ by heating for 12 h at 353 K under a He flow (10 ml/min), with the reusability test continued over 4 cycles.

C_2_H_2_/C_2_H_4_ = 50:50, v/v:

 The adsorbent (388 mg) (in the column Ø 6 mm × 400 mm) was activated at 423 K for 120 min under a He flow (20 ml/min). The column containing the adsorbent was then cooled to 298 K, whereupon a C_2_H_2_/C_2_H_4_ gas mixture (50:50, v/v) was introduced at a flow rate of 1.0 ml/min.

### Calculation of dynamic adsorption capacity

The amount of gas adsorbed *i* (*q_i_*) was calculated from the breakthrough curves using the following equation:

$$q_{i}=\frac{C_{i}V}{m}\times \int_{0}^{t}\left(1-\frac{F}{F_{0}}\right)\mathrm{dt}$$

where *q_i_* is the equilibrium adsorption capacity of gas *i* (cm^3^/g); *C_i_* is the feed gas concentration; *V* is the volumetric feed flow rate (ml/min); *t* is the adsorption time (s); *F_0_* and *F* are the inlet and outlet gas molar flow rates, respectively; and *m* is the mass of the adsorbent (g).

### DFT calculations

First-principles DFT calculations were performed with the Dmol^3^ module of Materials Studio [60,61]. DFT calculations were performed to provide the optimized structures and energies for the interaction of C_2_H_2_ and C_2_H_4_ with the frameworks of the COFs. Perdew–Burke–Ernzerhof (PBE) exchange-correlation functionals under the generalized gradient approximation (GGA) with the double-ξ numerical polarization (DPN) basis set were used within the Dmol^3^ program package in MS software [62]. Since the whole unit cell of each COF was too large, smaller primitive cells were used in the calculations. The tolerances of energy, gradient, and displacement convergence were 1.0 × 10^−5^ hartree, 2 × 10^−3^ hartree/Å, and 5 × 10^−3^ Å, respectively. The dispersion correction (DFT) was incorporated into calculations of the single-point energy, where the energy cutoff was 400.0 eV and the self-consistent field (SCF) tolerance was 1.0 × 10^−6^ eV/atom. The binding energies (Δ*E*_bind_) for the adsorbed structures consisting of a primitive cell with C_2_H_2_ and C_2_H_4_ were calculated by Δ*E*_bind_ = *E*_gas_ + *E*_COF_ − *E*_complex_, where *E*_complex_, *E*_gas_, and *E*_COF_ are the total energies of complex of gas with COFs, C_2_H_2_ and C_2_H_4_ gases, and the COFs at the optimized geometries, respectively.

### GCMC simulations

GCMC simulations were carried out to model the adsorption of C_2_H_2_ and C_2_H_4_ on COF-1, COF-2, and COF-3 using the sorption module at 298 K and 101.0 kPa (fugacity). A single-unit cell was used. The simulation box was kept rigid, and periodic boundary conditions were applied in all three dimensions. Metropolis method and the COMPASS force field were used. A total of 1 × 10^6^ equilibration steps and 1 × 10^7^ production steps were set. The Lennard–Jones and electrostatic interactions were combined to describe gas–gas and gas–framework interactions, respectively. The cutoff of 12.5 Å was employed for Lennard–Jones interaction, and electrostatic interaction was described by Ewald summation.

### MD simulations

MD simulations were performed to analyze the diffusion behavior between gases and COFs, with the simulations being carried out using the BIOVIA Materials Studio software package. The energy and geometry of the total systems of adsorbates and adsorbents obtained from GCMC calculation were optimized using the Forcite module. The universal force field (UFF) was applied for all optimizations [63]. Ewald and atom-based integration methods were applied for modeling and calculation of the electrostatic and van der Waals energy potentials at constant temperatures. In order to reach the equilibrium state, canonical ensemble (NVT) was initially applied for 500 ps on the systems. Micro-canonical ensemble (NVE) was applied on systems for 5 ps to reach equilibrium. (N, V, T, and E represent atomic number, volume, temperature, and energy, respectively.)

The RDFs *g*(*r*) representing the density distribution of characteristic atoms of adsorbate molecules around a given atom within the adsorbent framework were calculated using the following equation [64]:

$$g\left(r\right)=\frac{1}{\rho_{0}}\frac{n\left(r\right)}{V}\approx \frac{1}{\rho_{0}}\frac{n\left(r\right)}{4\pi r^{2}\delta r}$$

where *ρ_0_* and *n(r)* are the number of characteristic atoms of one unit volume and of the spherical shell of radius *r* to *r* + *δr*, respectively. *V* ≈ 4*πr^2^δr* is the volume of the spherical shell of the thickness *δr* at a distance *r* from the given atom. The RDFs were obtained by the Forcite module with a cutoff of 12.5 Å and an interval *δr* of 0.02 Å.

## Data Availability

The data are available from the authors upon a reasonable request.
