# Supplementary material for: Single-Molecule Traps in Covalent Organic Frameworks for Selective Capture of C2H2 from C2H4-Rich Gas Mixtures
Source: Research (Wash D C). 2024 Aug 26;7:0458. doi: 10.34133/research.0458 (PMC11345538; doi:10.34133/research.0458)
Supplement: Supplementary 1 — Figs. S1 to S28 Tables S1 to S10 [file research.0458.f1.docx]

Supplementary Materials for

Single-molecule Traps in Covalent Organic Frameworks for Selective Capture of C_2_H_2_ from C_2_H_4_-rich Gas Mixtures

Yilun Zhou^a^, Yinghui Xie^a^, Xiaolu Liu^a^, Mengjie Hao^a^, Zhongshan Chen^a^, Hui Yang^a,^*, Geoffrey I. N. Waterhouse^b^, Shengqian Ma^c,^*, and Xiangke Wang^a,^*

^a^College of Environmental Science and Engineering, North China Electric Power University, Beijing 102206, P.R. China.

^b^MacDiarmid Institute for Advanced Materials and Nanotechnology, School of Chemical Sciences, The University of Auckland, Auckland 1142, New Zealand.

^c^Department of Chemistry, University of North Texas, Denton, Texas 76201, United States.

| **Table S1.** Physical size and boiling point of C_2_H_2_ and C_2_H_4_. | | | | |
| --- | --- | --- | --- | --- |
| Molecule | Molecular dimension (Å) | | | Boiling point (K) |
|  | x | y | z |  |
| C_2_H_2_ | 5.5 | 3.3 | 3.3 | 188.4 |
| C_2_H_4_ | 4.8 | 4.1 | 3.2 | 169.4 |

| **Table S2.** Fractional atomic coordinates for the unit cell of COF-1. | | | |
| --- | --- | --- | --- |
| Pawley refined, *R*_wp_=4.19%, *R*_p_=3.24%  P-6, a=b=23.29 Å, c=3.49 Å, α=β=90° and γ=120° | | | |
| Atom | x | y | z |
| C1 | 0.3034 | 0.70942 | 0 |
| C2 | 0.25896 | 0.63607 | 0 |
| C3 | 0.42047 | 0.80803 | 0 |
| O4 | 0.27759 | 0.74367 | 0 |
| C5 | 0.48004 | 0.54948 | 0 |
| C6 | 0.43606 | 0.48043 | 0 |
| C7 | 0.45976 | 0.43592 | 0 |
| C8 | 0.52782 | 0.45821 | 0 |
| C9 | 0.57223 | 0.52693 | 0 |
| C10 | 0.54898 | 0.57213 | 0 |
| C11 | 0.59752 | 0.64192 | 0 |
| N12 | 0.63654 | 0.69783 | 0 |
| N13 | 0.45653 | 0.59665 | 0 |
| N14 | 0.54906 | 0.40961 | 0 |
| C15 | 0.59428 | 0.30585 | 0 |
| C16 | 0.62071 | 0.25932 | 0 |
| C17 | 0.61728 | 0.42445 | 0 |
| O18 | 0.5344 | 0.28205 | 0 |
| H19 | 0.47242 | 0.82325 | 0 |
| H20 | 0.3831 | 0.45918 | 0 |
| H21 | 0.42448 | 0.38323 | 0 |
| H22 | 0.62508 | 0.54678 | 0 |
| H23 | 0.49314 | 0.64622 | 0 |
| H24 | 0.51058 | 0.36075 | 0 |
| H25 | 0.65521 | 0.47603 | 0 |

| **Table S3.** Fractional atomic coordinates for the unit cell of COF-2. | | | |
| --- | --- | --- | --- |
| Pawley refined, *R*_wp_=5.02%, *R*_p_=3.96%  P-6/M, a=b=23.25 Å, c=3.49 Å, α=β=90° and γ=120° | | | |
| Atom | x | y | z |
| C1 | 0.59401 | 0.28921 | 0 |
| C2 | 0.63756 | 0.25909 | 0 |
| O3 | 0.53392 | 0.25377 | 0 |
| C4 | 0.61523 | 0.19233 | 0 |
| C5 | 0.52447 | 0.0695 | 0 |
| C6 | 0.45599 | 0.02416 | 0 |
| C7 | 0.5684 | 0.04436 | 0 |
| N8 | 0.54659 | 0.13962 | 0 |
| H9 | 0.65261 | 0.17783 | 0 |
| H10 | 0.42108 | 0.04209 | 0 |
| H11 | 0.62147 | 0.07624 | 0 |
| H12 | 0.50862 | 0.15084 | 0 |

| **Table S4.** Fractional atomic coordinates for the unit cell of COF-3. | | | |
| --- | --- | --- | --- |
| Pawley refined, *R*_wp_=4.37%, *R*_p_=3.45%  P-6/M, a=b=22.70 Å, c=3.45 Å, α=β=90° and γ=120° | | | |
| Atom | x | y | z |
| C1 | 0.26219 | 0.6211 | 0 |
| C2 | 0.30789 | 0.59641 | 0 |
| C3 | 0.18863 | 0.57215 | 0 |
| N4 | 0.14279 | 0.58995 | 0 |
| C5 | 0.45746 | 0.52996 | 0 |
| C6 | 0.42995 | 0.45875 | 0 |
| C7 | 0.47096 | 0.42933 | 0 |
| H8 | 0.28931 | 0.54175 | 0 |
| H9 | 0.17611 | 0.51838 | 0 |
| H10 | 0.37461 | 0.42706 | 0 |
| H11 | 0.4466 | 0.37393 | 0 |

| **Table S5.** Comparison of the low-pressure C_2_H_2_ uptake capacities of various COFs at 298 K. | | | |
| --- | --- | --- | --- |
| COF | C_2_H_2_ uptake (cm^3^/g) | | |
|  | 0.01 bar | 0.1 bar | 1 bar |
| NKCOF-11-ABC | 2.91 | 20.60 | 67.50 |
| NKCOF-62 | 1.56 | 11.99 | 52.81 |
| PAF-110 | 3.32 | 16.12 | 49.50 |
| COF-1 | 7.97 | 38.23 | 110.01 |

| **Table S6.** Comparison of reported COFs and MOFs for C_2_H_2_ adsorption at 298 K and 0.01 bar. | |
| --- | --- |
| Material | C_2_H_2_ uptake at 298 K and 0.01 bar (cm^3^/g) |
| AlFSIX-Cu-TPBDA (ZNU-8) | 7.62 |
| IITKGP-30a | 0.76 (295 K) |
| Zr-TCA | 0.59 |
| In-L6-IPA | 7.08 |
| NOTT-300 | 4.34 (293 K) |
| Zn_4_O(NTB)_2_ | 3.63 |
| FJU-112a | 4.55 (296 K) |
| [Zn(bdc)_0.5_(mtrz)] | 0.94 |
| CAU-10H | 1.57 |
| CAU-23 | 4.89 |
| CPL-1 | 0.5 |
| CPL-1-NH_2_ | 3.55 |
| COF-1 | 7.97 |

| **Table S7.** Comparison of reported COFs and MOFs for C_2_H_2_ adsorption and corresponding Q_st_ values under ambient conditions. | | |
| --- | --- | --- |
| Material | C_2_H_2_ uptake (cm^3^/g) | Q_st_ C_2_H_2_ (kJ/mol) |
| NKCOF-11-ABC | 68 | 30.8 |
| PAF-110 | 49.952 | 38.4 |
| AlFSIX-Cu-TPBDA (ZNU-8) | 113.12 | 27.2 |
| Na@COFECUT-1 | 89.7 | 7.68 |
| TpPa-NO_2_ | 63.73 | 42.57 |
| TP-ND | 53 | 32.8 |
| TP-IQD | 46 | 33.8 |
| PAF-120 | 50.848 | 37.5 |
| M’MOF-3a | 42.56 | 25 |
| SIFSIX-3-Zn | 81.536 | 31 |
| UTSA-100a | 95.648 | 22 |
| COF-1 | 110.008 | 35.95 |

| **Table S8.** Fitted parameters for the dual-site Langmuir-Freundlich (DSLF) equation describing C_2_H_2_ and C_2_H_4_ adsorption on COF-1 at 273 K and 298 K. | | | | |
| --- | --- | --- | --- | --- |
| Parameter | C_2_H_2_ | | C_2_H_4_ | |
| Temperature | 273 K | 298 K | 273 K | 298 K |
| *q_sat, A_* (mmol/g) | 4.60313 | 3.49446 | 2.16333 | 2.51878 |
| *q_sat, B_* (mmol/g) | 13.0617 | 7.65593 | 4.52980 | 3.45618 |
| *b_A_* (kPa^-1^) | 0.20233 | 0.08903 | 0.00248 | 0.00013 |
| *b_B_* (kPa^-1^) | 0.01069 | 0.01025 | 0.03600 | 0.02057 |
| t_A_ | 1.36022 | 1.25388 | 1.12967 | 0.69655 |
| t_B_ | 1.44728 | 1.26141 | 1.19319 | 1.13218 |
| R^2^ | 0.99999 | 0.99999 | 0.99999 | 0.99999 |

| **Table S9.** Fitted parameters for the dual-site Langmuir-Freundlich (DSLF) equation describing C_2_H_2_ and C_2_H_4_ adsorption on COF-2 at 273 K and 298 K. | | | | |
| --- | --- | --- | --- | --- |
| Parameter | C_2_H_2_ | | C_2_H_4_ | |
| Temperature | 273 K | 298 K | 273 K | 298 K |
| *q_sat, A_* (mmol/g) | 5.10764 | 3.45698 | 2.31339 | 3.88896 |
| *q_sat, B_* (mmol/g) | 2.47116 | 2.38059 | 2.26322 | 0.28182 |
| *b_A_* (kPa^-1^) | 0.09543 | 0.06121 | 0.04946 | 0.00555 |
| *b_B_* (kPa^-1^) | 0.00019 | 0.00233 | 0.00028 | 0.05929 |
| t_A_ | 1.38331 | 1.30941 | 1.09058 | 1.02066 |
| t_B_ | 0.57827 | 0.88574 | 0.55871 | 0.92718 |
| R^2^ | 0.99997 | 0.99997 | 0.99992 | 0.99996 |

| **Table S10.** Fitted parameters for the dual-site Langmuir-Freundlich (DSLF) equation describing C_2_H_2_ and C_2_H_4_ adsorption on COF-3 at 273 K and 298 K. | | | | |
| --- | --- | --- | --- | --- |
| Parameter | C_2_H_2_ | | C_2_H_4_ | |
| Temperature | 273 K | 298 K | 273 K | 298 K |
| *q_sat, A_* (mmol/g) | 2.18733 | 1.38019 | 1.23020 | 1.63410 |
| *q_sat, B_* (mmol/g) | 4.72485 | 1.70321 | 2.35742 | 0.04599 |
| *b_A_* (kPa^-1^) | 0.10065 | 0.00085 | 0.11275 | 0.03776 |
| *b_B_* (kPa^-1^) | 0.00120 | 0.06063 | 0.00234 | 0.22024 |
| t_A_ | 1.33849 | 0.74200 | 1.22132 | 1.20062 |
| t_B_ | 0.86200 | 1.22987 | 0.84584 | 0.67566 |
| R^2^ | 0.99999 | 0.99999 | 0.99999 | 0.99992 |


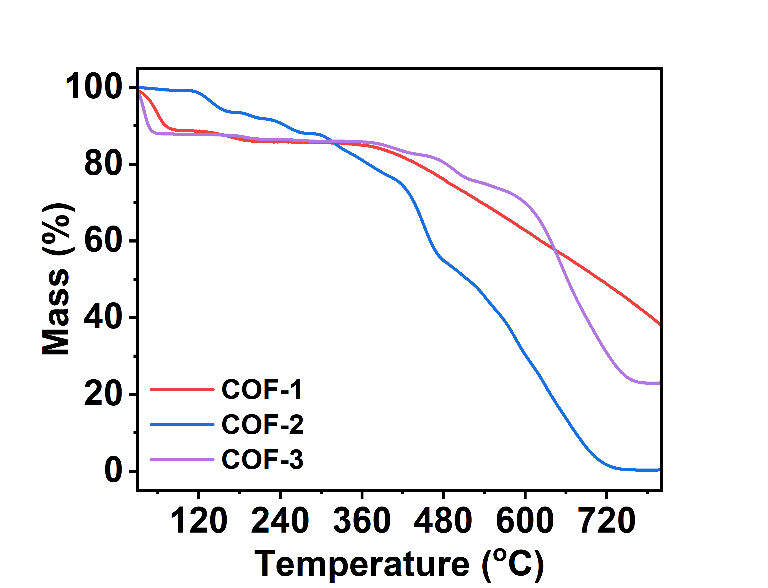


**Fig. S1.** TGA curves for COF-1, COF-2 and COF-3.

**
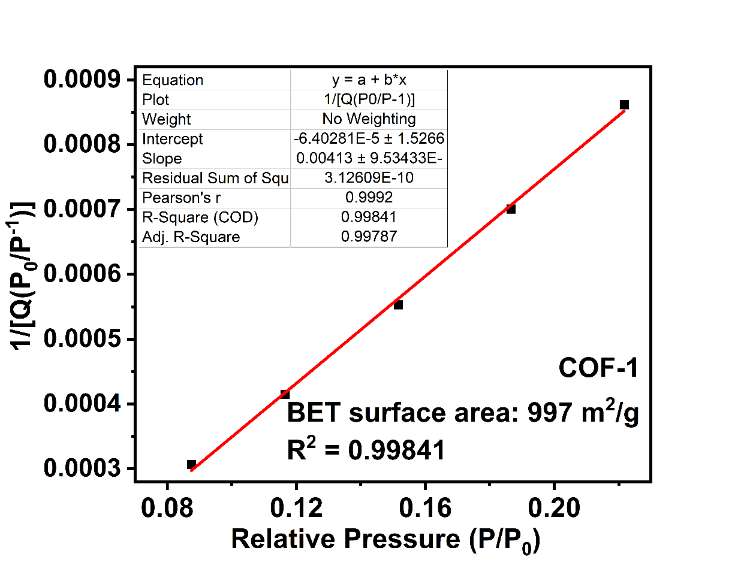
**

**Fig. S2.** BET equation plot for COF-1.


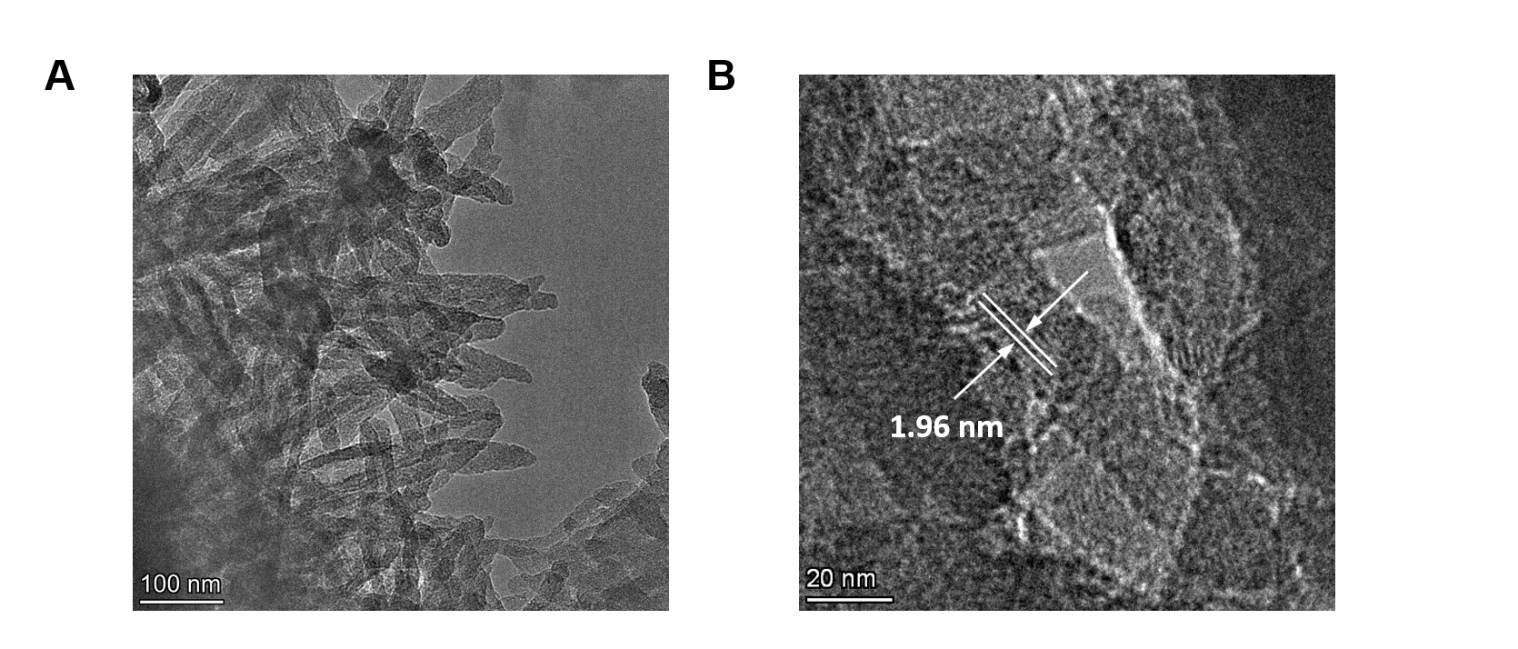


**Fig. S3.** TEM (A) and HRTEM (B) images for COF-1.


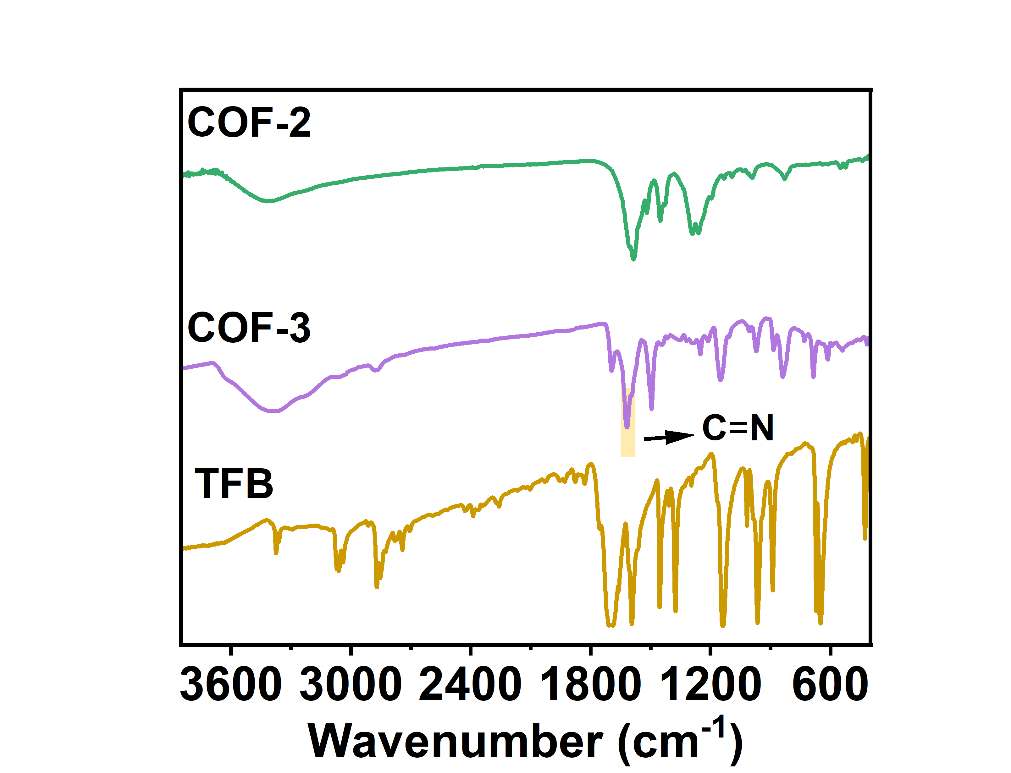


**Fig. S4.** FT-IR transmittance spectra of COF-2, COF-3 and the TFB linker.


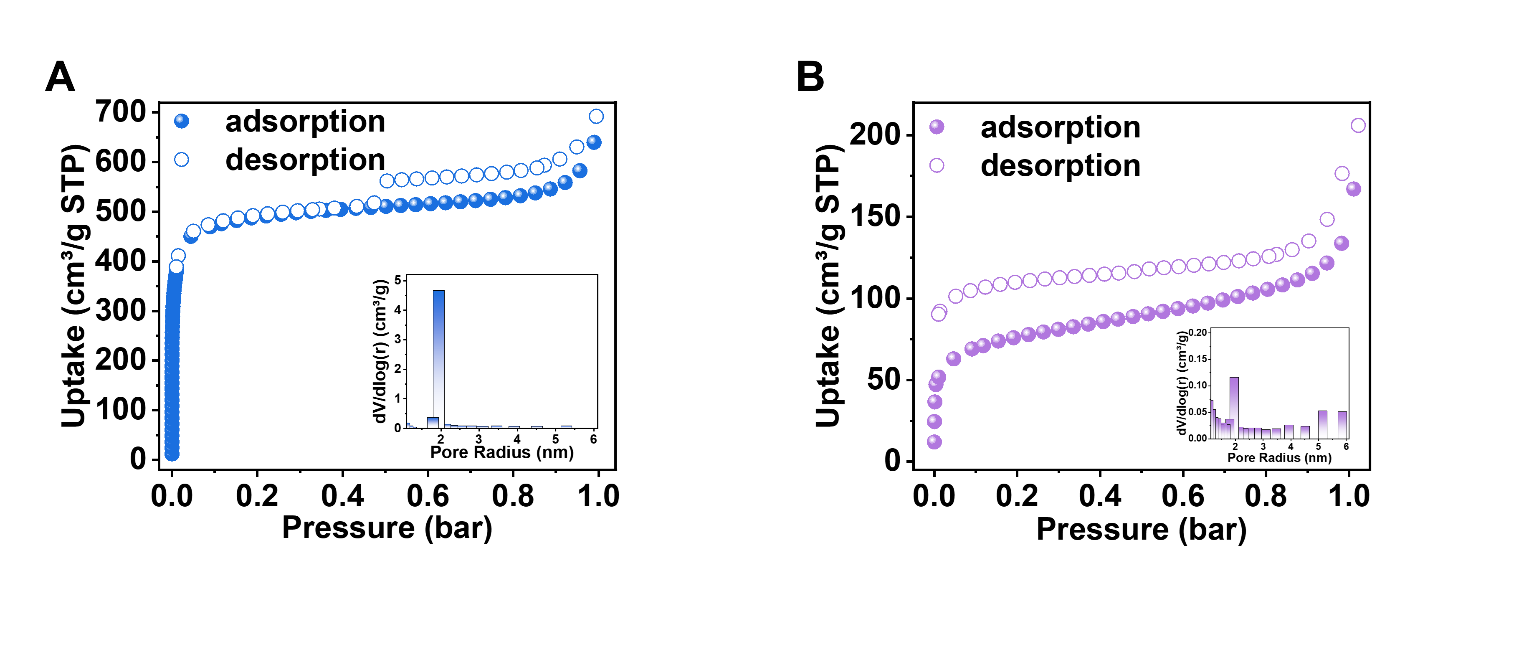


**Fig. S5.** Nitrogen adsorption and desorption isotherms collected at 77 K for (A) COF-2 and (B) COF-3. The insets show the corresponding pore size distribution of each COF.


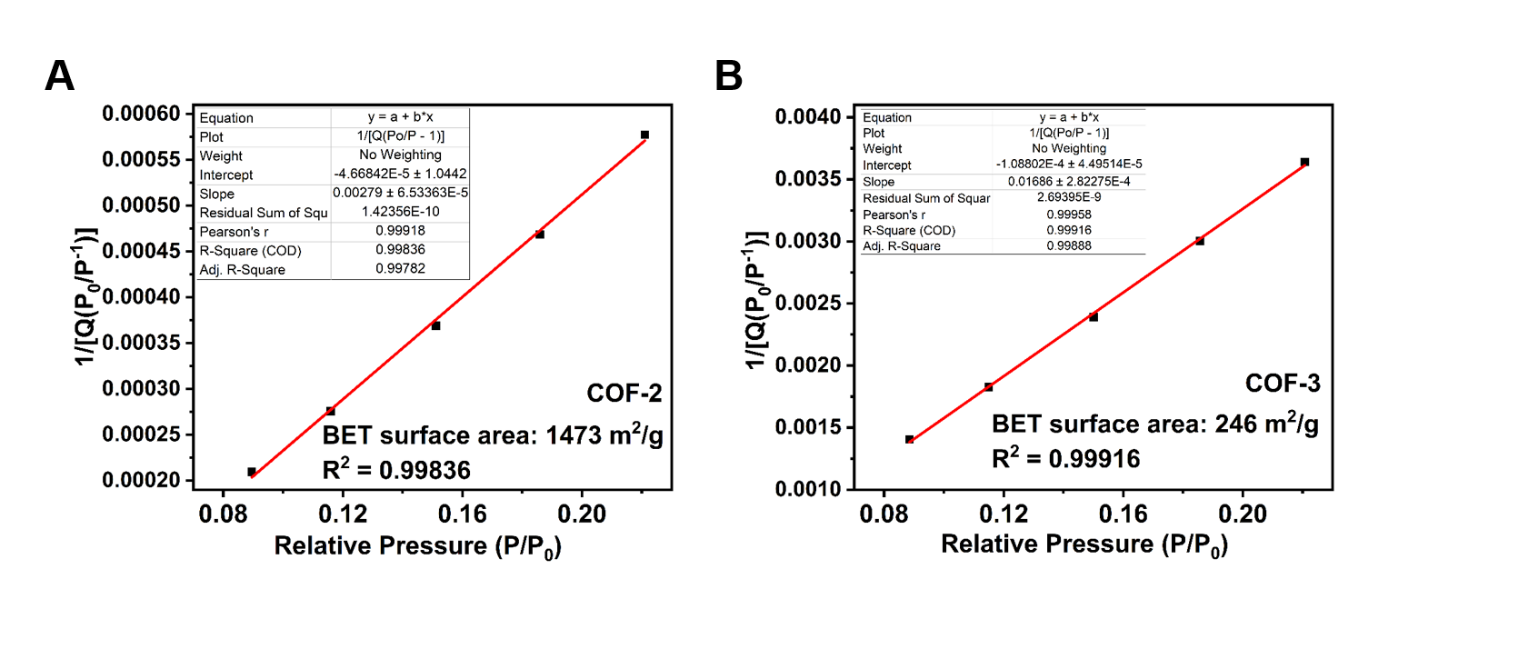


**Fig. S6.** BET equation plots for (A) COF-2 and (B) COF-3.


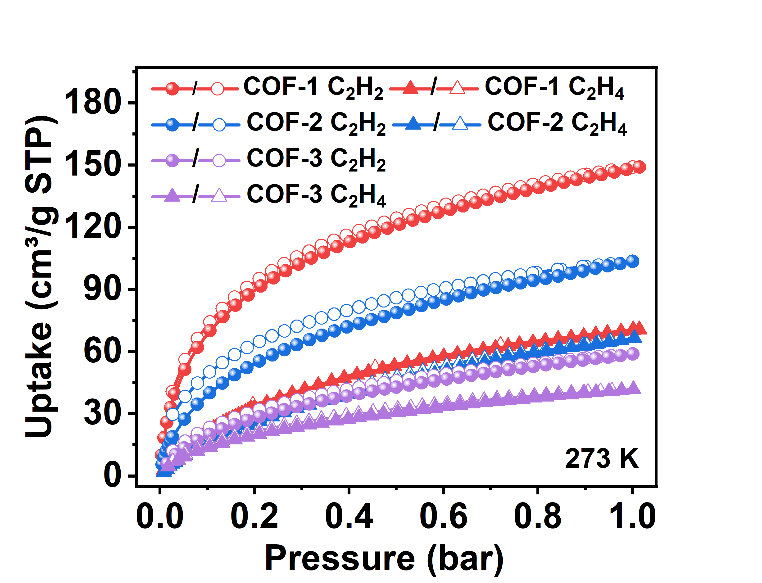


**Fig. S7.** Single-component C_2_H_2_ and C_2_H_4_ adsorption (filled symbols) and desorption (open symbols) isotherms of COF-1 (red), COF-2 (blue) and COF-3 (purple) at 273 K.


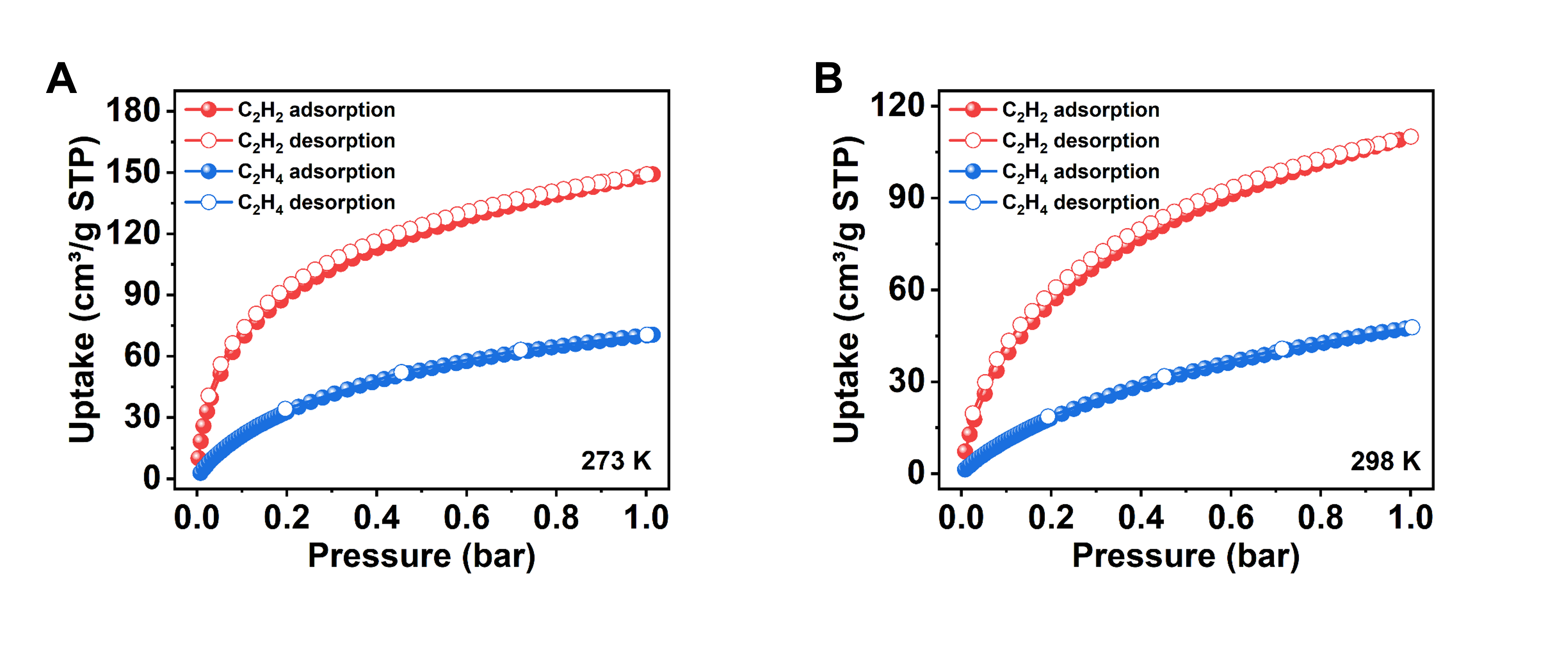


**Fig. S8.** Single-component C_2_H_2_ and C_2_H_4_ adsorption-desorption isotherms of COF-1 at (A) 273 K and (B) 298 K.


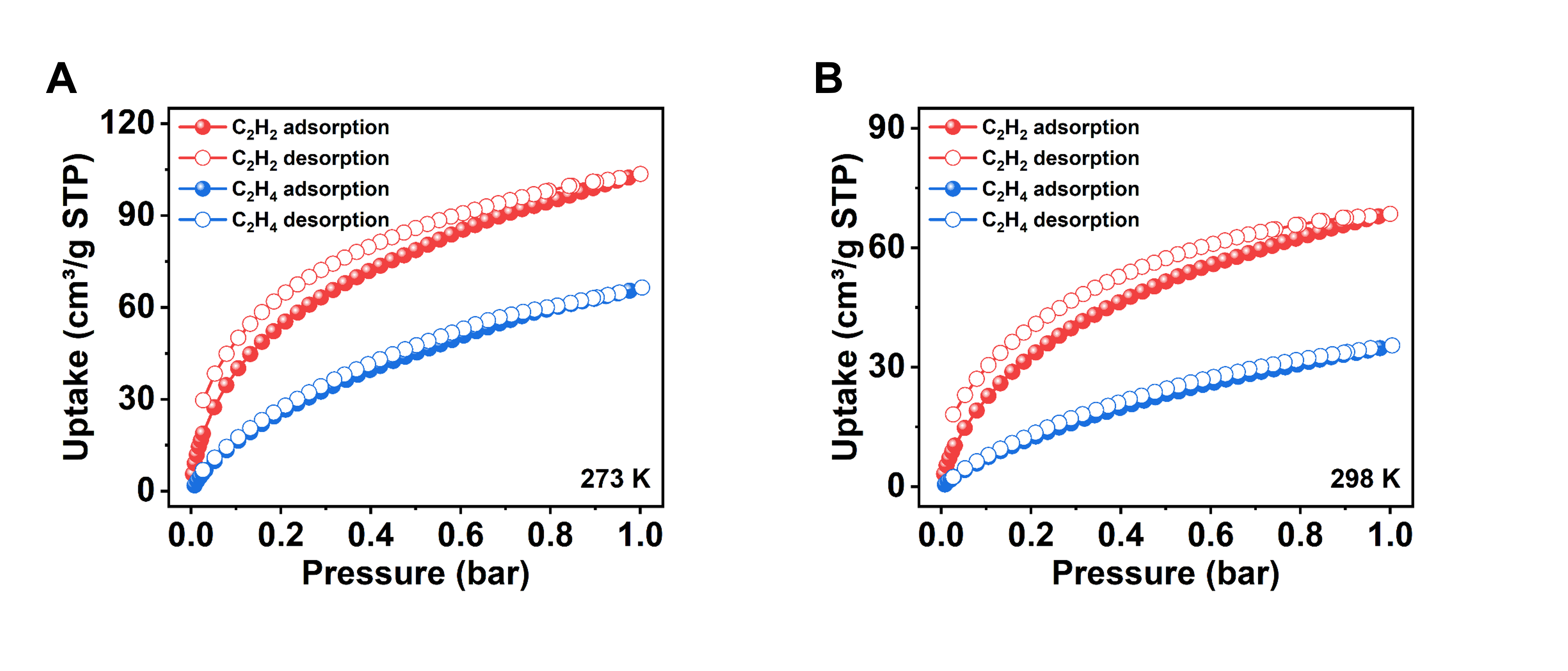


**Fig. S9.** Single-component C_2_H_2_ and C_2_H_4_ adsorption-desorption isotherms of COF-2 at (A) 273 K and (B) 298 K.


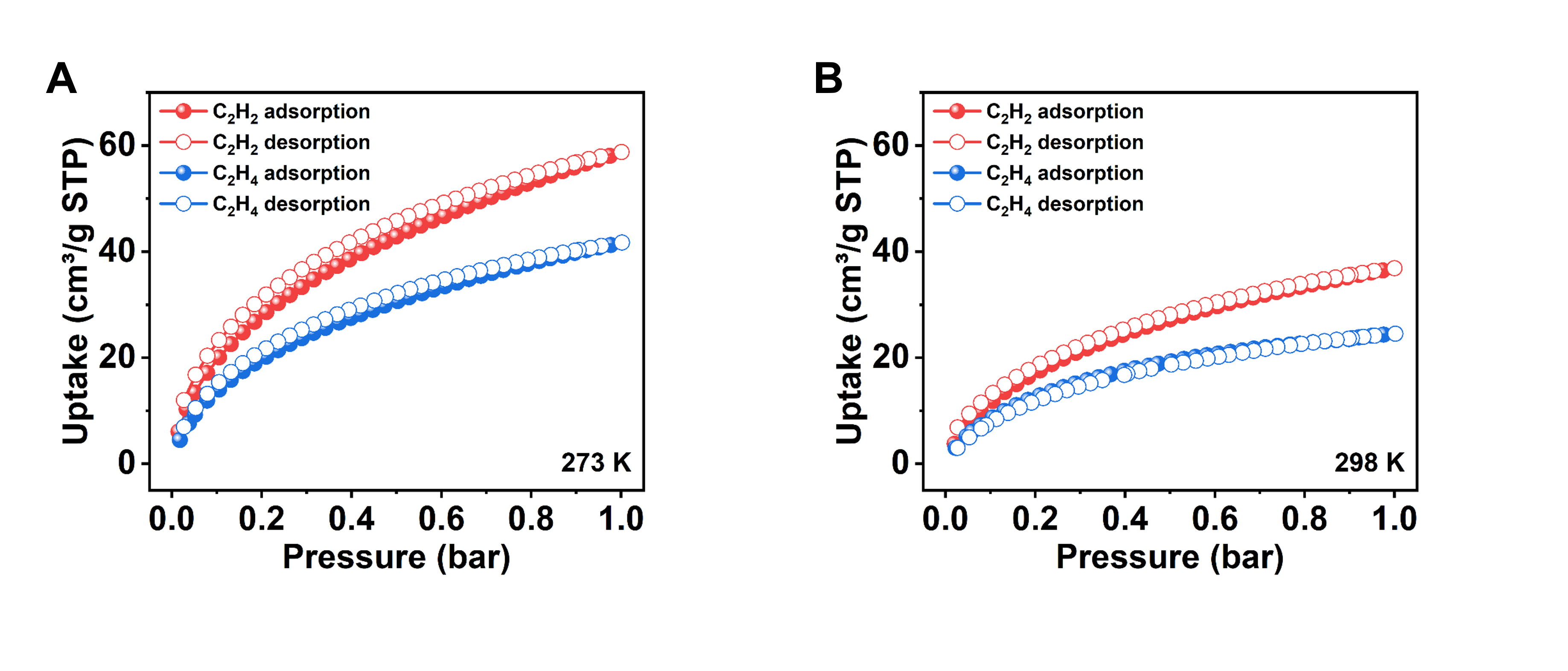


**Fig. S10.** Single-component C_2_H_2_ and C_2_H_4_ adsorption-desorption isotherms of COF-3 at (A) 273 K and (B) 298 K.


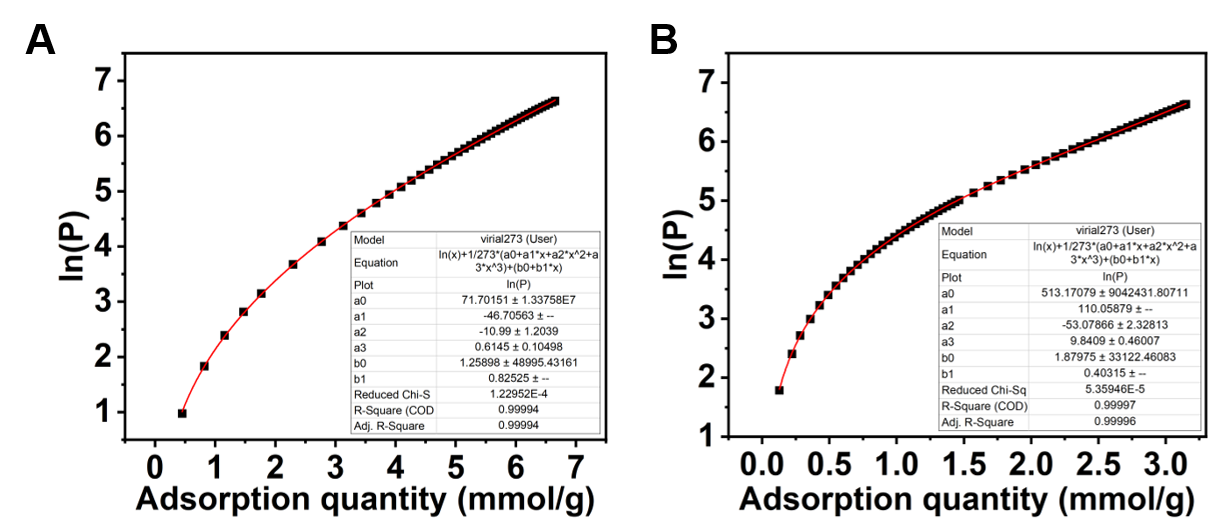


**Fig. S11.** Virial fitting plots for COF-1 at 273 K. (A) C_2_H_2_ and (B) C_2_H_4_.


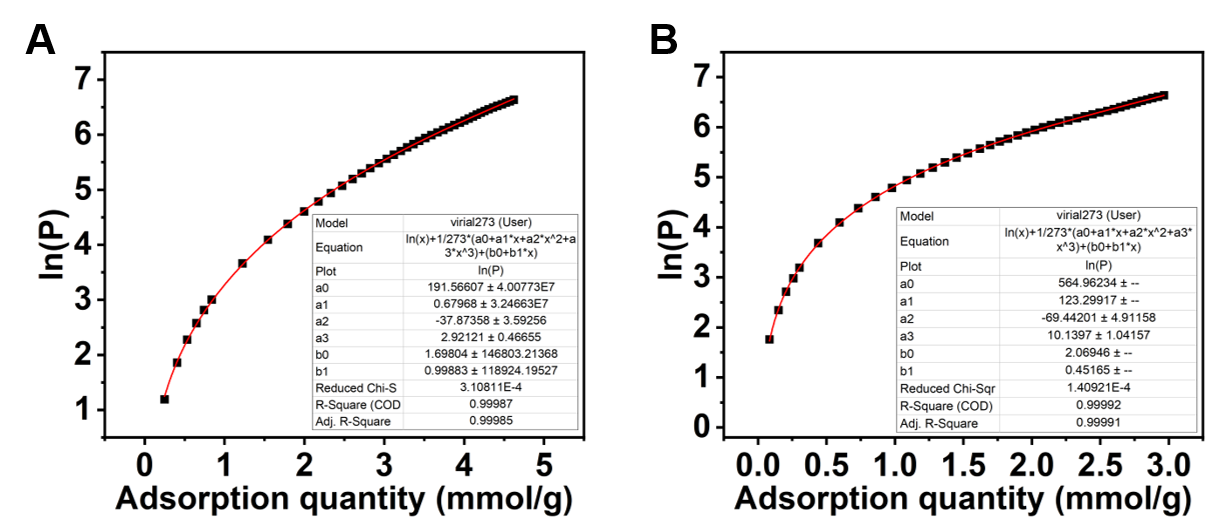


**Fig. S12.** Virial fitting plots for COF-2 at 273 K. (A) C_2_H_2_ and (B) C_2_H_4_.


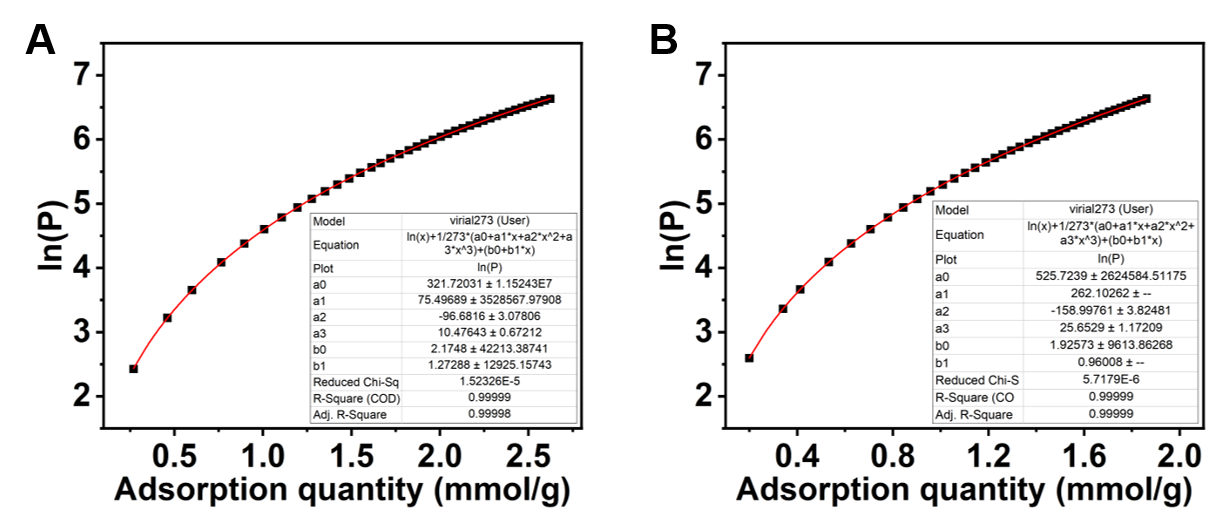


**Fig. S13.** Virial fitting plots for COF-3 at 273 K. (A) C_2_H_2_ and (B) C_2_H_4_.


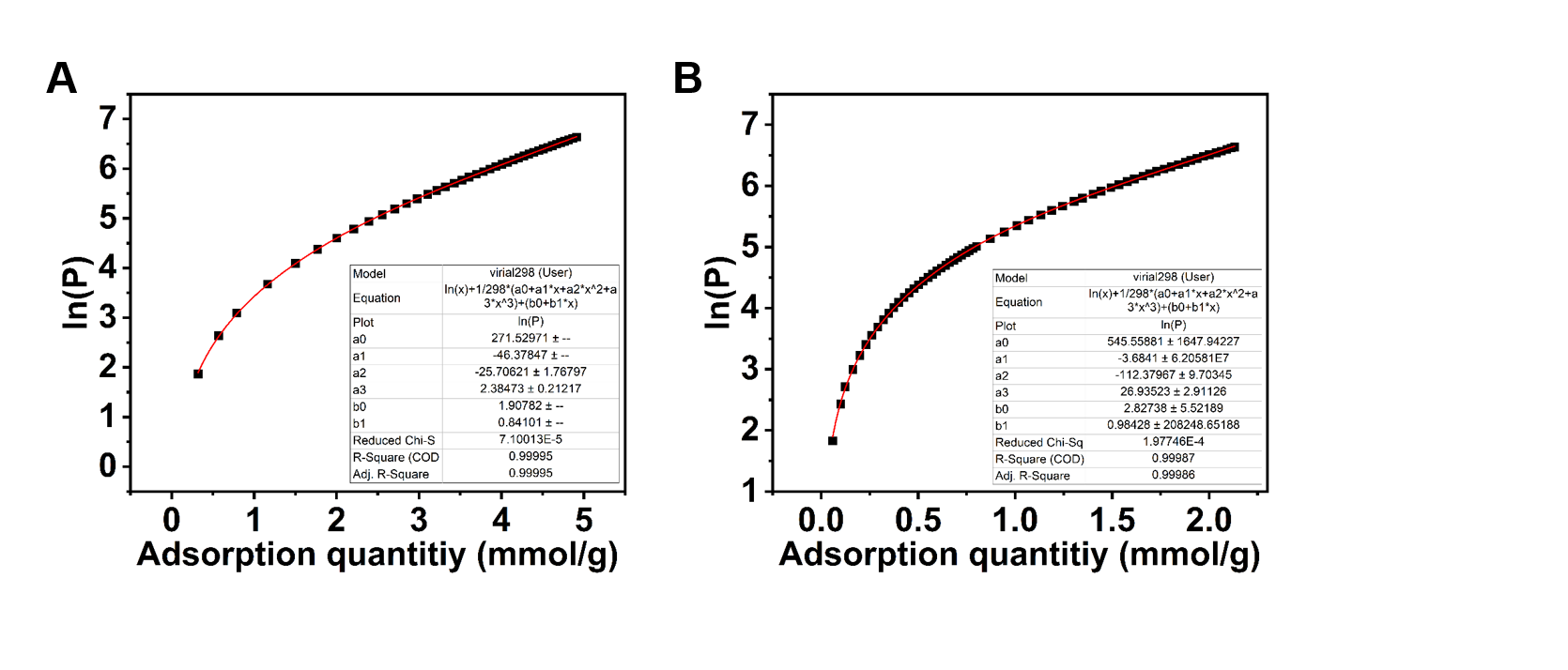


**Fig. S14.** Virial fitting plots for COF-1 at 298 K. (A) C_2_H_2_ and (B) C_2_H_4_.


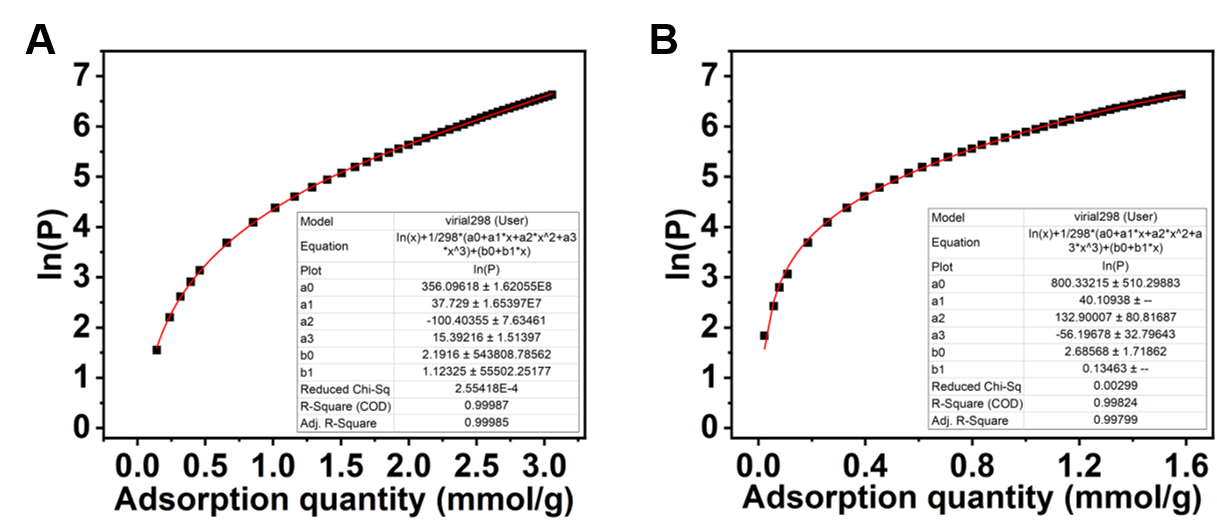


**Fig. S15.** Virial fitting plots for COF-2 at 298 K. (A) C_2_H_2_ and (B) C_2_H_4_.


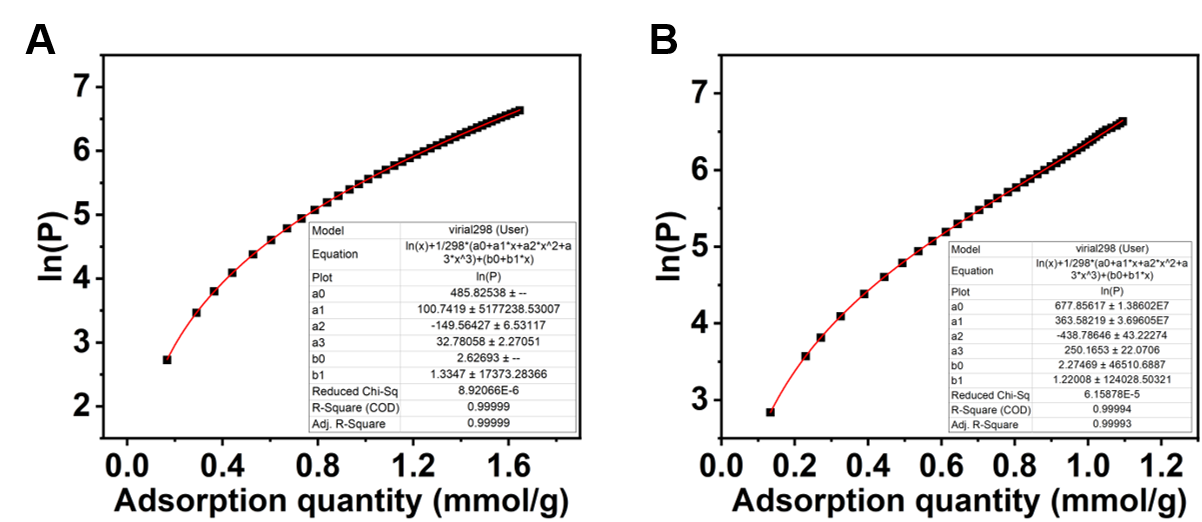


**Fig. S16.** Virial fitting plots for COF-3 at 298 K. (A) C_2_H_2_ and (B) C_2_H_4_.


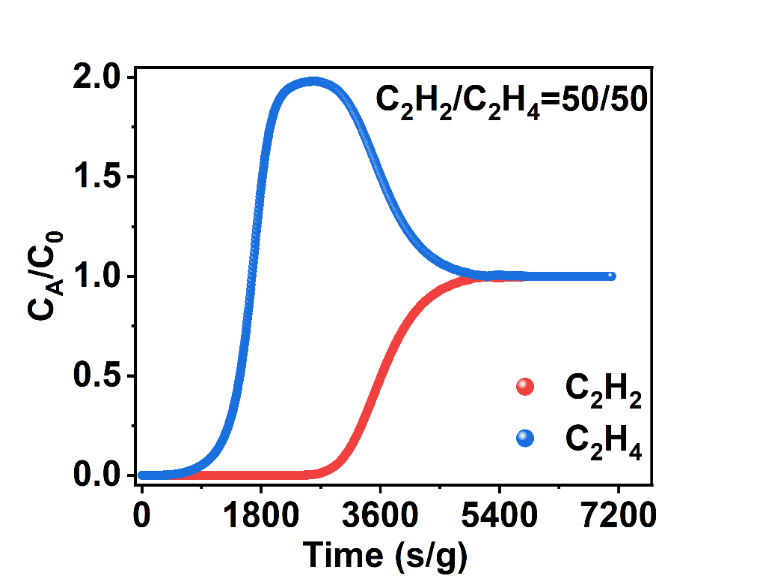


**Fig. S17.** Experimental breakthrough curves for a C_2_H_2_/C_2_H_4_ (1:1, v/v) gas mixture using COF-1 at 298 K (1 bar). The gas flow rate was 1.0 mL/min.


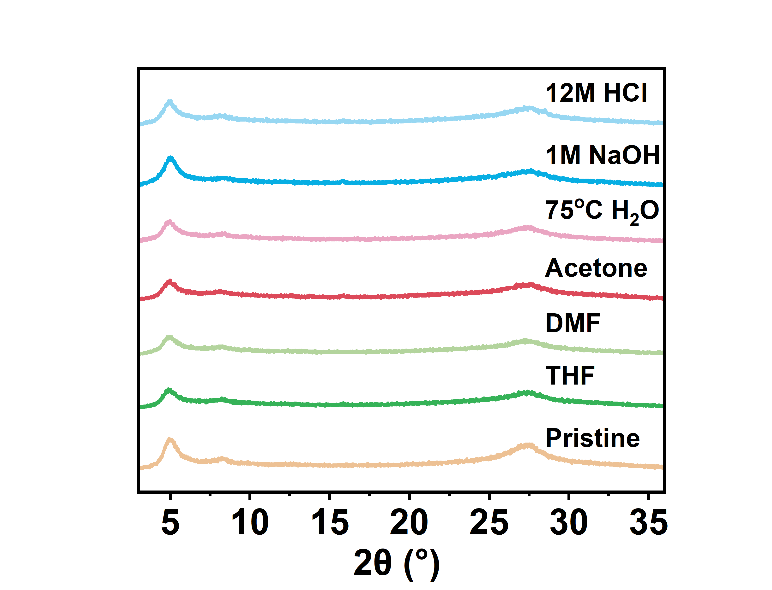


**Fig. S18.** PXRD patterns of COF-1 following various treatments for 24 h.


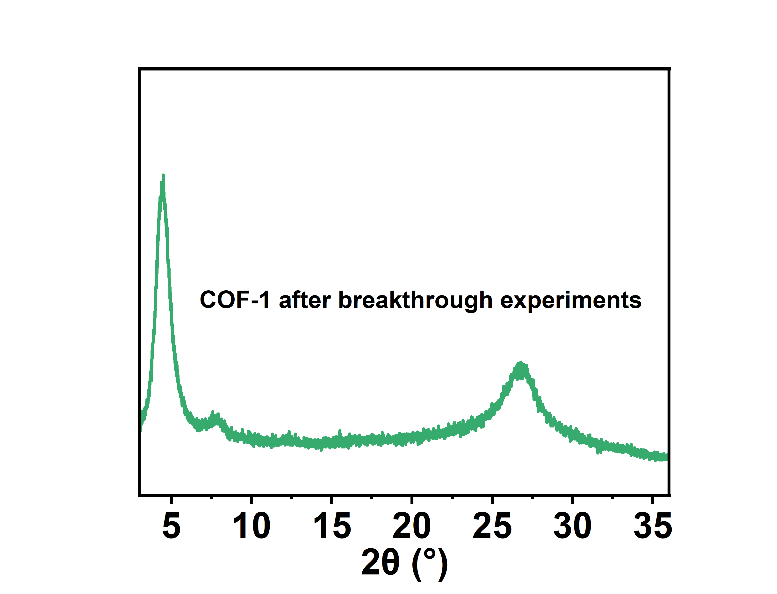


**Fig. S19.** PXRD pattern of COF-1 after breakthrough experiments.


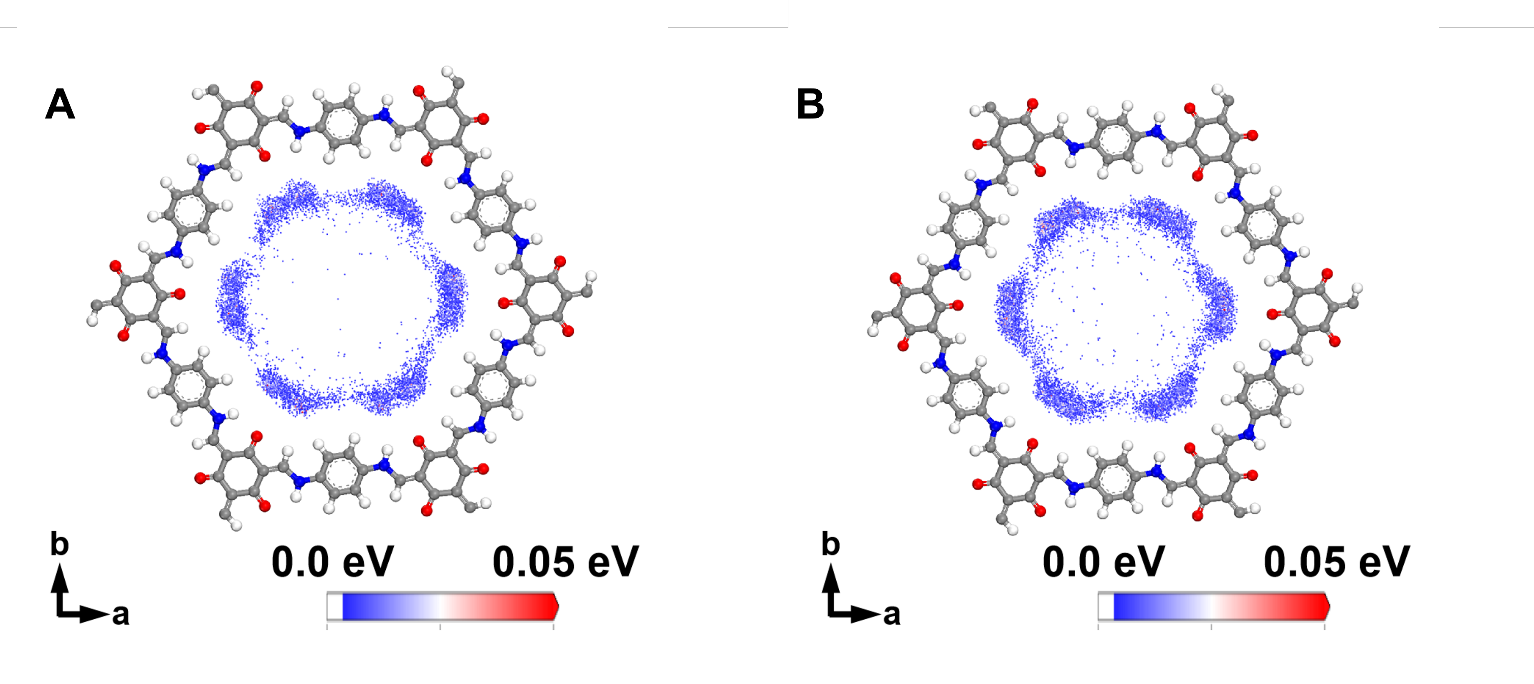


**Fig. S20.** Computational simulations showing the density of (A) C_2_H_2_ and (B) C_2_H_4_ on COF-2 at 298 K and 1 bar.


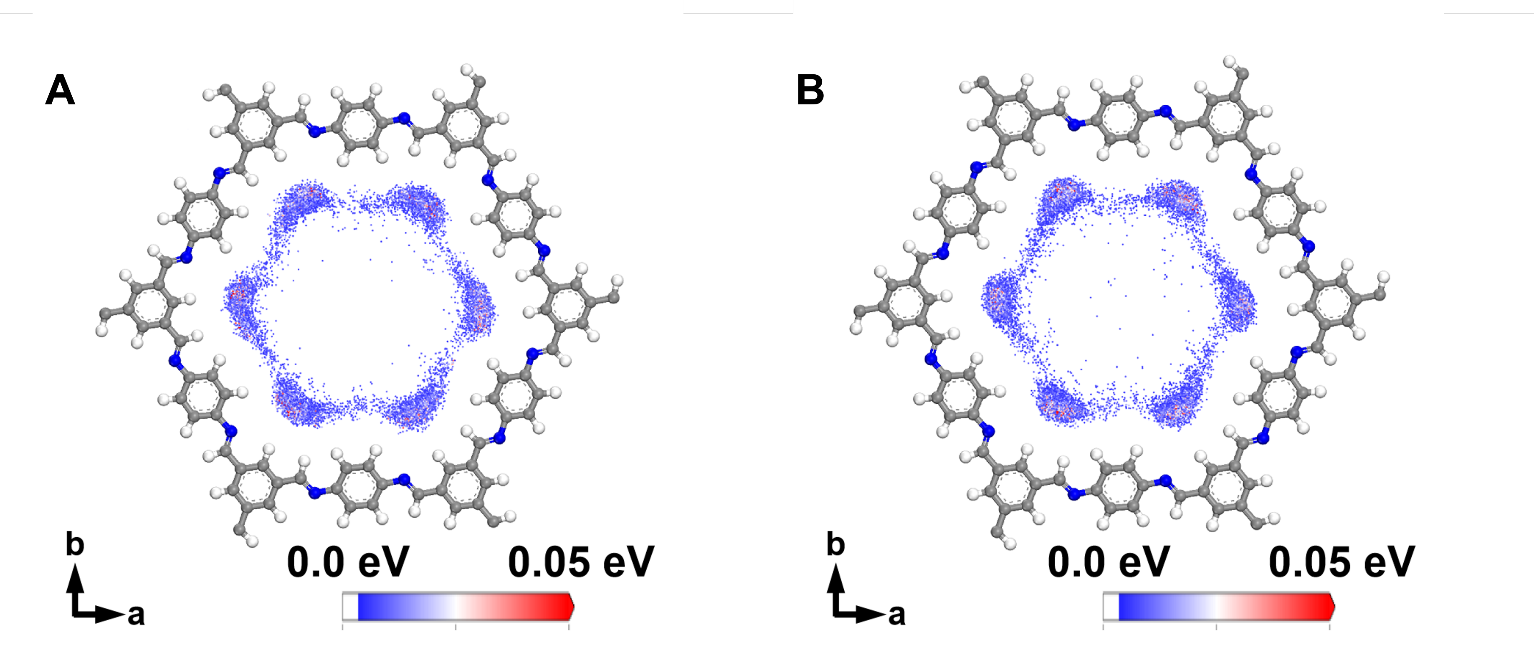


**Fig. S21.** Computational simulations showing the density of (A) C_2_H_2_ and (B) C_2_H_4_ on COF-3 at 298 K and 1 bar.


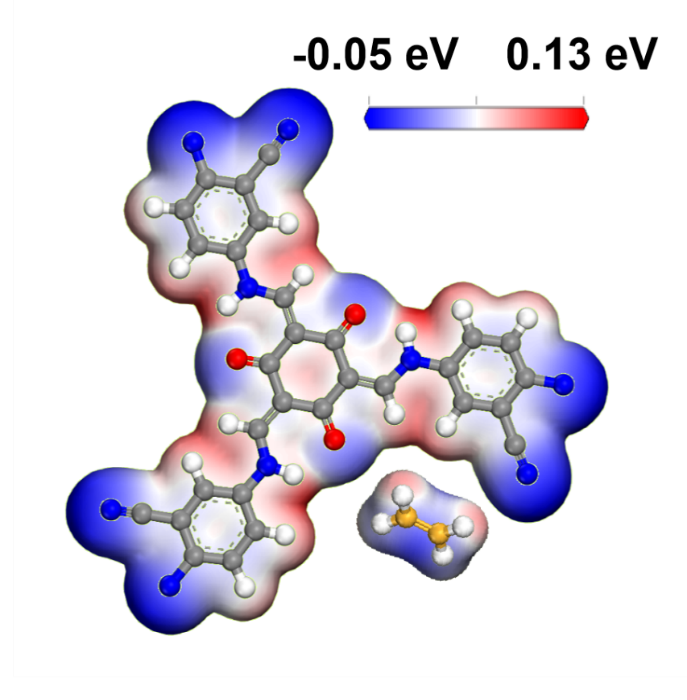


**Fig. S22.** Electrostatic potentials of site I in COF-1 with C_2_H_4_.


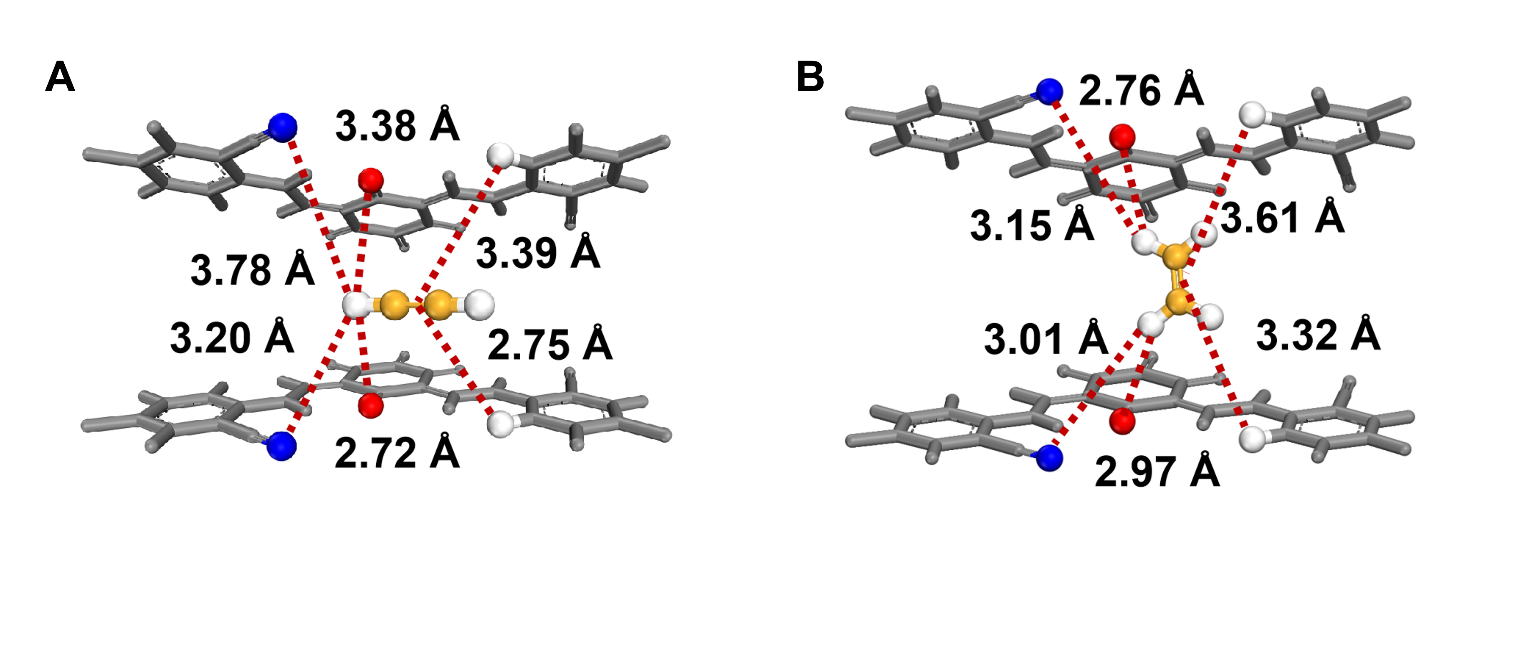


**Fig. S23.** DFT optimized gas adsorption configuration and bonding distances of (A) C_2_H_2_ and (B) C_2_H_4_ in COF-1 (site II).


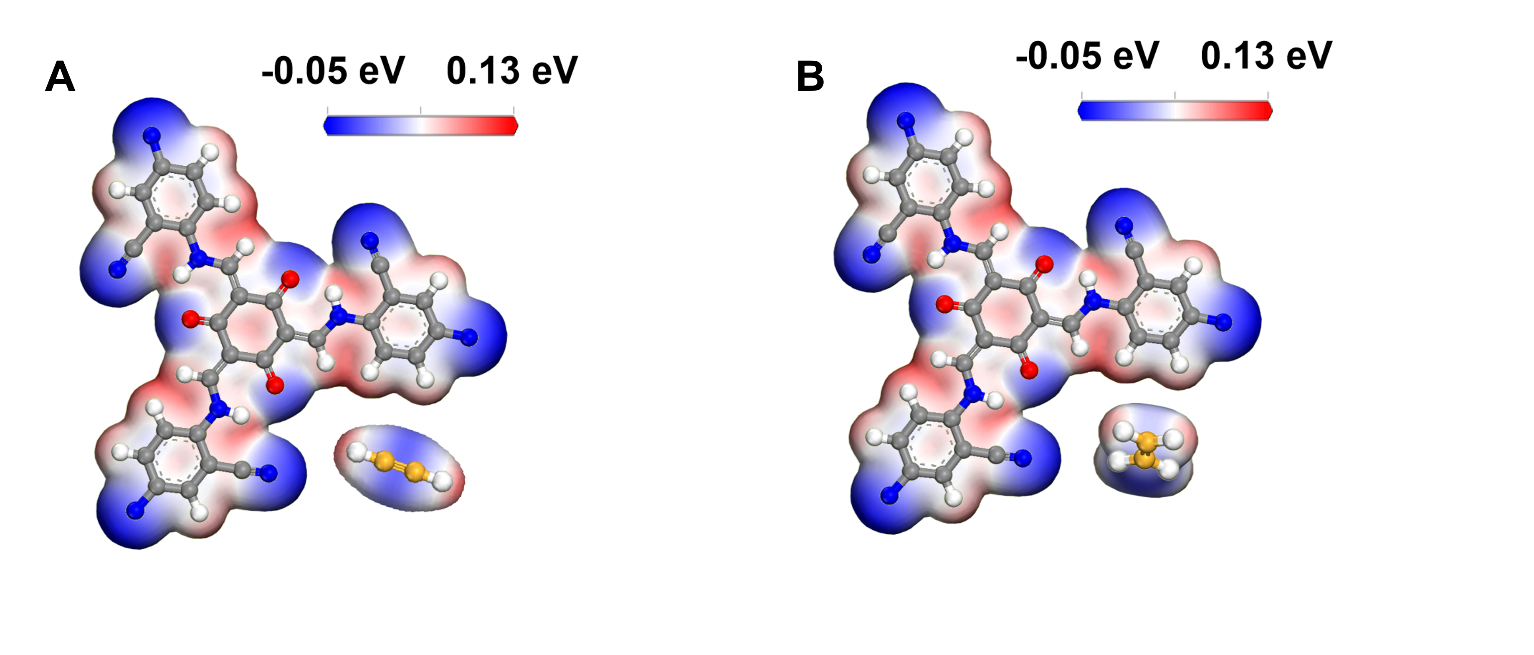


**Fig. S24.** Electrostatic potentials of site II in COF-1 with (A) C_2_H_2_ and (B) C_2_H_4_.


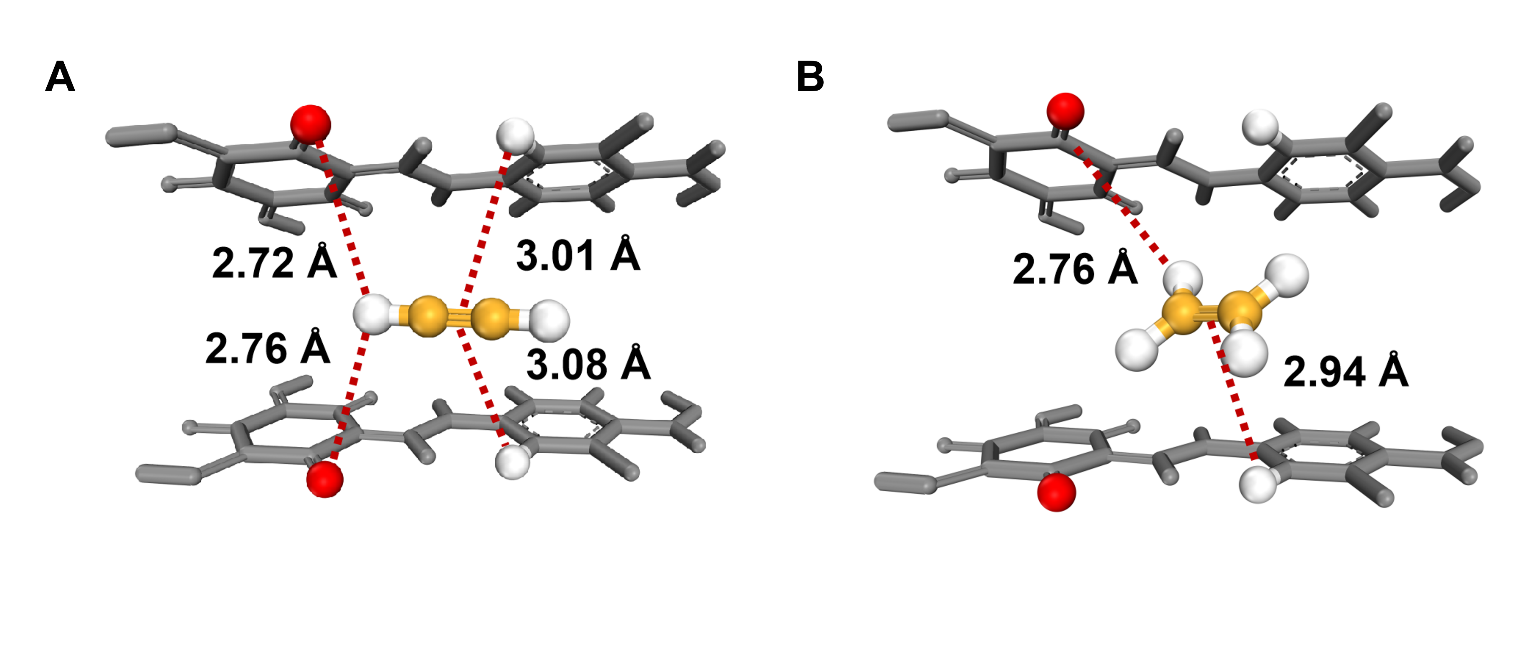


**Fig. S25.** DFT optimized gas adsorption configuration and bonding distances of (A) C_2_H_2_ and (B) C_2_H_4_ in COF-2.


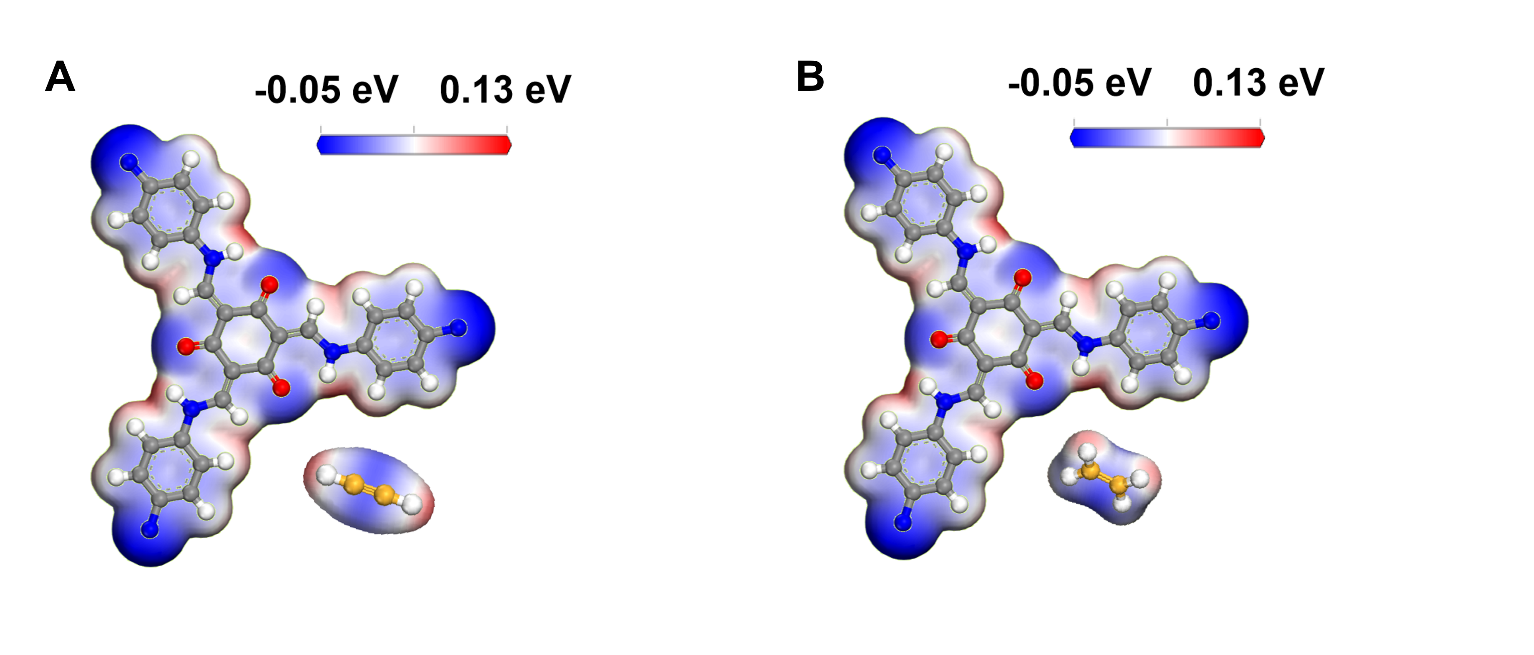


**Fig. S26.** Electrostatic potentials of the sorption site in COF-2 with (A) C_2_H_2_ and (B) C_2_H_4_.


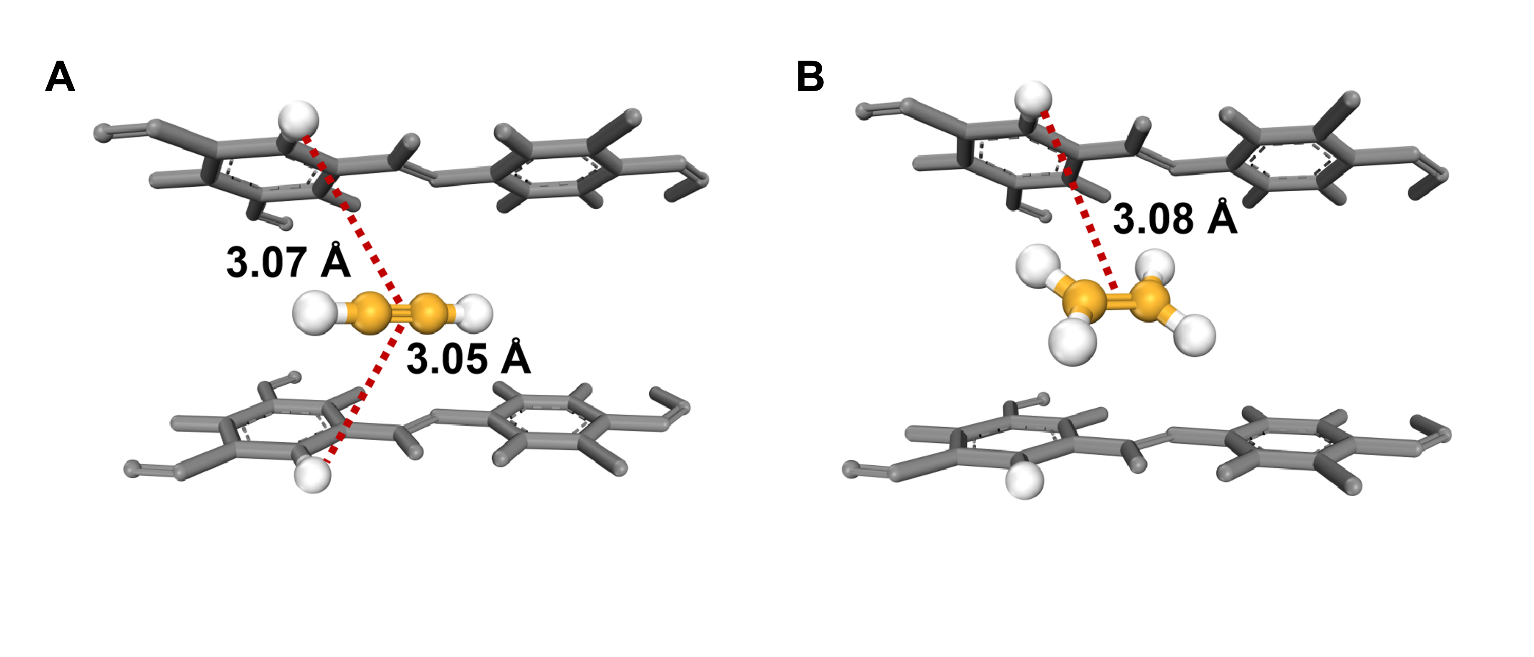


**Fig. S27.** DFT optimized gas adsorption configuration and bonding distances of (A) C_2_H_2_ and (B) C_2_H_4_ in COF-3.


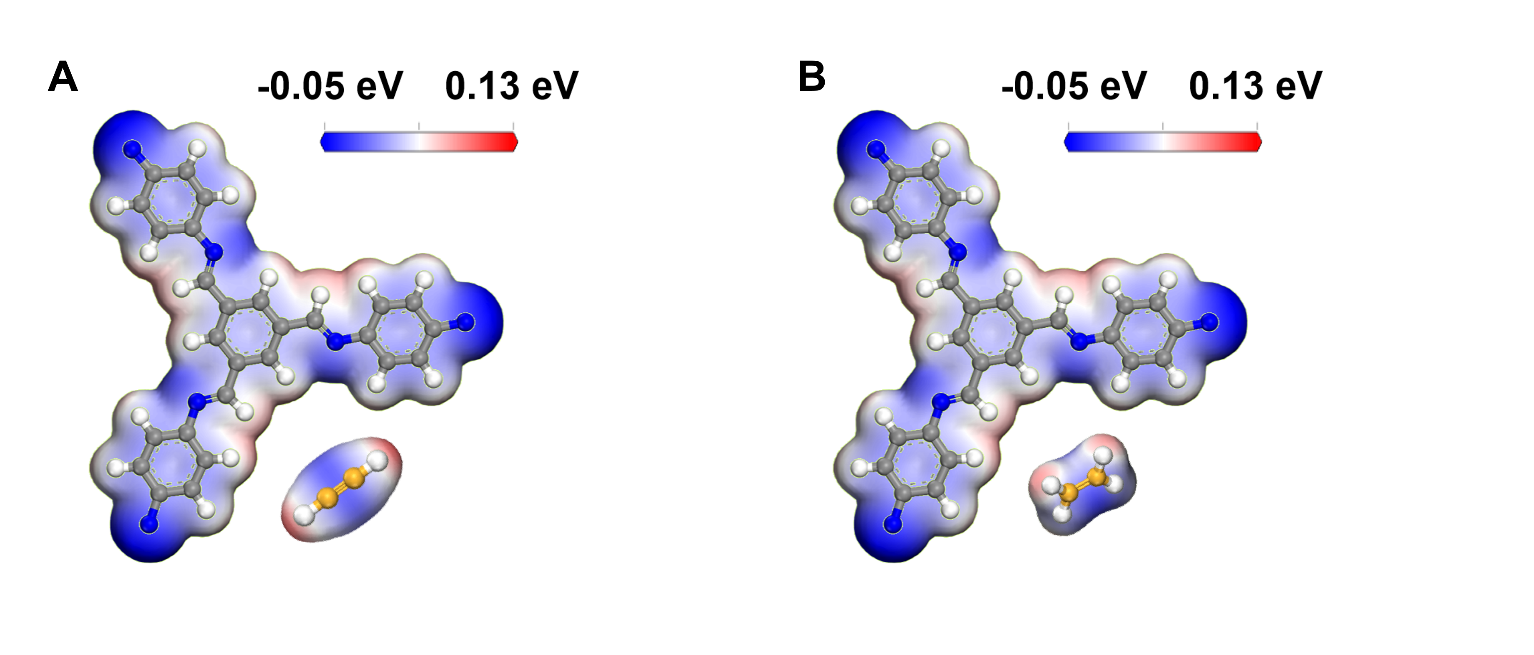


**Fig. S28.** Electrostatic potentials of the sorption site in COF-3 with (A) C_2_H_2_ and (B) C_2_H_4_.
